# Effects of Synthetic Tetronamides and Methylated Denigrins on Bacterial Quorum Sensing and Biofilm Formation

**DOI:** 10.1021/acsomega.3c01729

**Published:** 2023-10-02

**Authors:** Sweta Roy, Jaime A. M. Acosta, Milandip Karak, Isabela Ramirez-Velez, Kohei Torikai, Dacheng Ren, Luiz C. A. Barbosa

**Affiliations:** † Department of Biomedical and Chemical Engineering, Syracuse University, Syracuse, New York 13244, United States; ‡ Department of Chemistry, Universidade Federal de Minas Gerais, Av. Pres. Antônio Carlos, 6627, Campus Pampulha, Belo Horizonte, MG CEP 31270-901, Brazil; § Chemical Technology School, Universidad Tecnológica de Pereira, Carrera 27 #10-02, Barrio Álamos, Risaralda, Pereira Código postal 660003, Colombia; ∥ Department of Chemistry, Faculty of Science, Kyushu University, 744 Motooka, Nishi-ku, Fukuoka 819-0395, Japan; ⊥ Faculty of Chemistry, National University of Uzbekistan named after Mirzo Ulugbek, 4 University Str., Tashkent 100174, Uzbekistan; ▲ Department of Biomedical and Chemical Engineering and Civil and Environmental Engineering and Biology, Syracuse University, Syracuse, New York 13244, United States

## Abstract

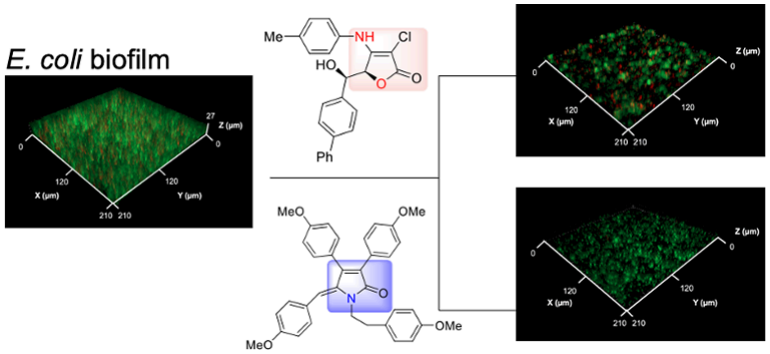

Detrimental biofilms of bacterial pathogens cause chronic infections with a high-level tolerance to antibiotics. To identify new control agents, we synthesized and tested a total of 14 tetronamides (including 5 new compounds) and 6 denigrin intermediates on the model species *Escherichia coli*. At a concentration of 50 μg/mL, two tetronamides and two methylated denigrins exhibited significant inhibitory effects against biofilm formation of *E. coli* RP437, e.g., by 60 and 94%, respectively. Structural analysis of the tested compounds revealed that *p*-methoxybenzylidene and *p-*methoxyphenethyl moieties of denigrins are important for biofilm inhibition, while the former group is also essential to the activity against quorum sensing (QS) via AI-2. Specifically, tetramethyldenigrin B has strong inhibitory effects against both *E. coli* biofilm formation and AI-2-mediated QS and thus provides a promising lead structure for designing better control agents. Consistently, tetramethyldenigrin B also showed inhibitory activity against biofilm formation of uropathogenic *E. coli*. Together, these findings provide new insights for the rational design of novel biofilm and QS inhibitors.

## Introduction

Developing new control agents is a priority for medicinal chemistry in combating bacterial infections.^1−4^ Reports from the World Health Organization show that antibiotic-resistant infections could cause 10 million deaths annually by 2050 if no new effective antibiotics are developed.^5^ The unremitting emergence of multidrug-resistant bacterial pathogens has motivated scientists to search for new sources of molecules against bacterial pathogens.^1^ One area that has attracted increasing attention is to target bacterial multicellular systems such as biofilms, which are consortia of microbial cells attached to a surface and enclosed in a self-produced extracellular matrix. The biofilm matrix consists of polysaccharides, proteins, and extracellular DNA and protects bacteria from a variety of physical, chemical, and biological stresses.^6^ For example, biofilms are up to 1000 times more tolerant to antibiotics than their planktonic counterparts.^7^ Thus, it is important to identify effective agents that can prevent biofilm formation. In contrast to conventional antibiotics that target bacterial growth, antibiofilm agents do not need to be bactericidal or growth inhibiting, reducing the chances of developing resistance. Another biofilm-associated target is quorum sensing (QS), a form of bacterial cell–cell communication via secreted signaling molecules (autoinducers)^8^ that allows bacteria to sense cell density and make physiological changes in response to environmental factors.^9,10^ The important role of QS in bacterial virulence motivated studies in the past three decades to search for QS quenching agents.^10−13^ It is envisaged that interception of these signaling processes will reduce the production of virulence factors and biofilm formation.^14^ Hence, the infection may be treated with lower doses of antibiotics or cleared by the host immune system.

**Figure 1:**
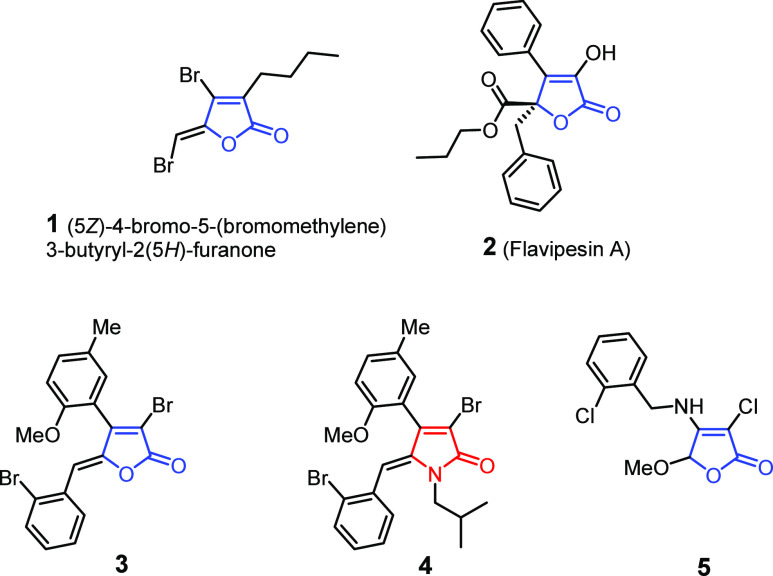
Natural QS inhibitor (**1**), antibiofilm agent (**2**), and synthetic compounds with antibiofilm (**3** and **4**) and antibacterial activities (**5**).

To date, a number of natural products and their synthetic analogues have been reported as QS inhibitors and antibiofilm agents.^15−29^ One class of the best studied natural antibiofilm agents to date are brominated furanones (e.g., compound **1**; Figure 1) derived from the red alga *Delisea pulchra*.^30^ These small molecules and their synthetic derivatives exhibited potent inhibitory activities against biofilm formation of many microbial pathogens such as *Escherichia coli*,^31,32^*Pseudomonas aeruginosa*,^33^*Streptococci spp*.,^34^*Staphylococcus epidermidis*,^34,35^*Salmonella enterica*,^36^ and sulfate-reducing bacteria.^37^ Studies have shown that these furanones could also reduce QS mediated by acyl-homoserine lactones (AHLs; AI-1) and AI-2 in Gram-negative bacteria.^38^ In contrast, flavipesin A (**2**), derived from an endophytic fungus, *Aspergillus flavipes*, demonstrated the antibiofilm activity against *Staphylococcus aureus* by decreasing the number of live cells embedded in the biofilm matrix, which indicates that flavipesin A could penetrate the biofilm matrix to kill the attached cells.^12^

Over the past years, motivated by the findings of butenolides as biofilm inhibitors, our groups have synthesized different analogues of natural butenolides and tested their antibiofilm activities.^39−43^ Among them, a rubrolide analogue **3** and its lactam derivative **4** reduced the biofilm formation of *P. aeruginosa* with IC_50_ values of 3.9 and 0.6 μg/mL, respectively.^40^

It is noteworthy to mention that 4-amino-substituted butenolides (e.g., compound **5**), also known as tetronamides, have significant antibiotic activities against a variety of Gram-positive and Gram-negative bacteria including multidrug-resistant *S. aureus*.^44−46^ We recently demonstrated that a series of tetronamide aldolates have potent inhibitory activities against bloom-forming cyanobacteria.^47^ To further understand the potential of tetronamides in microbial control, we aimed to synthesize new derivatives and test their biological activities against bacterial QS and biofilm formation.

Herein, we report the synthesis of five new tetronamides and their evaluation, along with recently reported analogues, against biofilm formation and QS of *E. coli*. This study also includes a comparative analysis of the effects of marine natural products denigrins A and B and their synthetic intermediates on QS.

## Results and Discussion

### Chemical Synthesis

**Figure 2:**
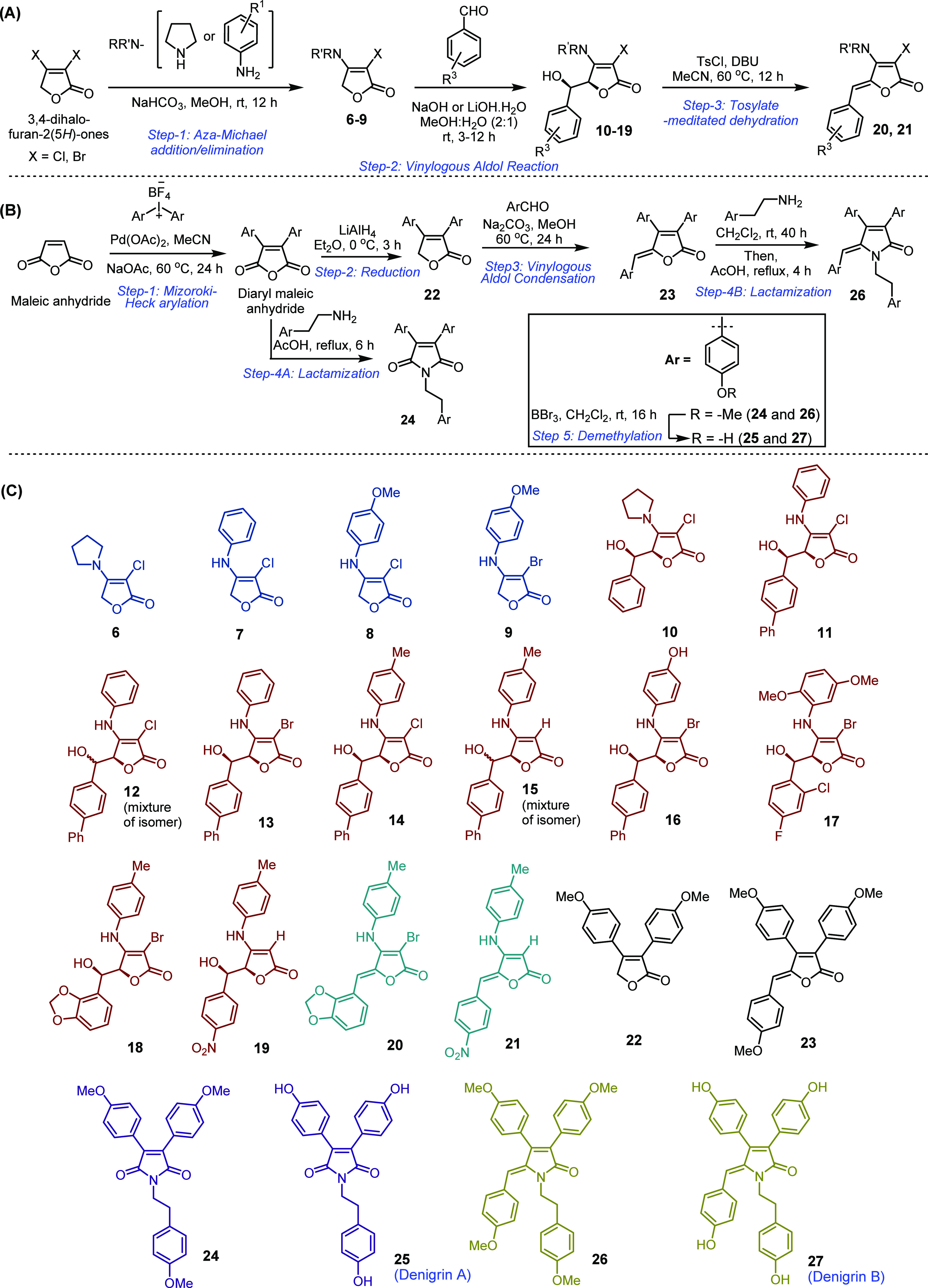
(A) Overview of the syntheses of tetronamide derivatives. Only the *syn* isomers are depicted for simplicity. The ESI contains detailed information on the *syn*/*anti* ratio and percentage of yields. (B) Overview of the syntheses of denigrins. (C) Structures of tetronamides and denigrins tested for antibiofilm activities.

In this study, compounds **6**–**27** (Figure 2) were prepared, with compounds **6**, **10**, **17**, **18**, and **20** being new. All compounds consist of a butenolide core and can be divided into two categories: synthetic tetronamides (compounds **6**–**21**) and natural denigrins and intermediates (compounds **22**–**27**).

All compounds were synthesized using previously reported procedures, as summarized in Figure 2A,B.^47−51^ In general, the tetronamides were prepared from commercially available 3,4-dihalofuran-2(5*H*)-ones, which were subjected to an *aza*-Michael addition/elimination reaction to afford tetronamide intermediates **6**–**9** (Figure 2A, step 1). These intermediates were treated with several substituted aldehydes via a vinylogous aldol reaction (VAR), followed by tosylate-mediated dehydration (Figure 2A, steps 2 and 3). The VAR produced mainly *syn*-aldol adducts with high yields and diastereoselectivity, achieved through *anti-syn* isomer interconversion.^48^ In most cases, we were able to purify the main *syn* product using chromatographic methods, except for compounds **11** and **15**, for which the isomers were not successfully separated. For compound **11**, we obtained a mixture of *syn-anti* isomers in a 45:55 ratio and labeled it as **12**.

The main goal of this work is to explore the potential of tetronamides for microbial control. To achieve this, we synthesized simple structures such as compounds **6** and **10** to determine whether the core structure (butenolide linked to a pyrrolidine moiety) with or without the γ-attached benzyl group would retain its activity. This structural simplification is a common strategy in this research area. We also prepared compounds **18** and **20** because many bioactive natural products have a methylenedioxy group attached to an aromatic ring.^52^ These two compounds expand the main structural feature of the tetronamide series being investigated. Compound **17** also fits into the main structural diversity of aromatic tetronamides and allows us to evaluate the influence of fluorine and chlorine on bioactivities as the effect of halogens on different bioactivities is well documented.

The natural products denigrins A and B (**25** and **27**) and their fully methylated precursors **24** and **26** were also evaluated in this work. A general synthetic route for these compounds is shown in Figure 2B. In short, the compounds were prepared starting from maleic anhydride, which was subjected to a Mizoroki–Heck arylation (Figure 2B, step 1), followed by reduction (Figure 2B, step 2) to produce the butanolide core **22**. Further reaction of **22** with *p*-methoxybenzaldehyde using a vinylogous aldol condensation (Figure 2B, step 3) yielded the γ-benzylidene intermediate **23**. In the next step (Figure 2B, step 4B), the reaction of **23** with 2-(4-methoxyphenyl)ethan-1-amine resulted in compound **26**. The reaction of diaryl maleic anhydride with 2-(4-methoxyphenyl)ethan-1-amine (Figure 2B, step 4A) produced compound **24**. Finally, borontribromide-mediated demethylation of **24** and **26** (Figure 2B, step 5) produced denigrins A and B, respectively.^27^

Full details of the synthesis experimental procedures along with spectroscopic (^1^H and ^13^C NMR, mass spectra) and physical data leading to characterization of all new compounds are available in the ESI. For the known compounds, the data are consistent with those previously published by our groups.^25−27^

### Antibiofilm Activity of Tetronamides

**Figure 3:**
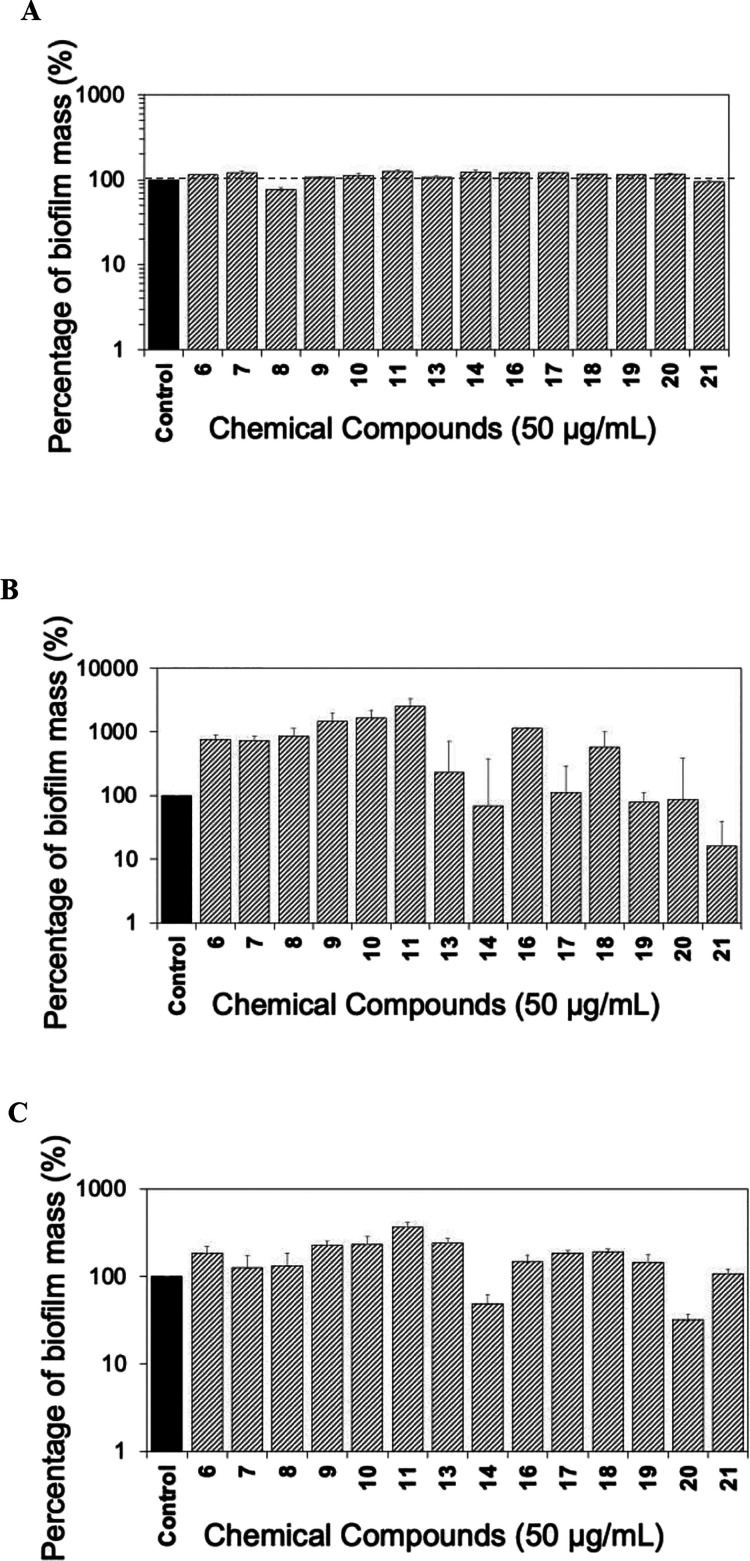
Crystal violet microtiter biofilm assay was used to evaluate the biofilm formation of *E. coli* RP437 when exposed to tetronamide derivatives and compared to untreated controls. The results were normalized by signals of sterile medium (LB or LB with 50 μg/mL of the compounds). (A) Total biomass based on OD_600_ measured to evaluate the toxicity of candidate compounds and normalized by the control as 100%. (B) Effects of the compounds on bacterial biofilm formation at the bottom of the well normalized by the control. (C) Effects of the chemical compounds on total biofilm formation (after addition of 95% ethanol; also includes the air–liquid interface biofilms) normalized by the control. Means ± SE are presented (*n* = 4).

In total, 14 tetronamide derivatives, which include four 4-amino-substituted butenolides (**6**–**9**), eight tetronamide aldolates (**10**, **11**, **13**, **14**, **16**–**19**), and two 5-alkylidene-4-amino-substituted butenolides (**20**–**21**), were tested for antibiofilm activities against *E. coli* RP437. Initially, a microtiter plate-based crystal violet assay was used to evaluate the bacterial planktonic growth and biofilm formation (Figure 3). In this assay, the growth inhibition was analyzed by means of total absorbance measured at an optical density at 600 nm (Figure 3A), while the effects on biofilm formation at the liquid–surface interface at the bottom of wells (Figure 3B) and the total biofilm formation (including biofilms at both the bottom of the well and the liquid–air interface) were measured based on absorbance at 560 nm after staining with crystal violet (Figure 3C). The biofilm at the bottom of the well (liquid–solid interface) is a submerged biofilm like those on submerged surfaces (e.g., an implanted medical device or the surface of pipe transferring liquid). In contrast, biofilms formed at the air–liquid interface involve bacterial response to oxygen gradient, as seen in some industrial systems.^53^ Both measurements provide valuable information for evaluating the activities of these compounds. Some compounds exhibited different effects on liquid–solid and air–liquid interface biofilms. The mechanism is unknown but likely involves complex interactions between genetic and physiological factors. It is beyond the scope of this study (effects on biofilm and QS) and will be part of our future work.

When tested at 50 μg/mL, the total growth of *E. coli* RP437 (OD_600_) was not significantly affected by the compounds, except for the chlorinated tetronamide **8**, showing a modest reduction (25%; *p* = 0.012, one-way ANOVA, followed by the Tukey test). In comparison, the tested compounds exhibited different effects on the *E. coli* biofilms. As shown in Figure 3B, the majority of compounds increased biofilm formation at the bottom of the well, while a few showed biofilm reduction. Similar effects were observed with total biofilm formation (Figure 3C). For example, the biphenyl derivatives **14** (64% biofilm reduction at the bottom of the well and 60% of the total biofilm) and the benzylidene derivatives **20** (18% biofilm reduction at the bottom of the well, but 72% reduction of the total biofilm) and **21** (78% at the bottom of the well), along with aldolate **19** (20% at the bottom of the well), were able to reduce *E. coli* RP437 biofilm formation at the bottom of the well and/or the total biofilm by 20–80% compared to the untreated control (Figure 3B,C). Since these compounds did not exhibit a significant effect on bacterial growth, the observed biofilm reduction can be attributed to factors related to biofilm formation rather than growth inhibition or killing effects like conventional antibiotics. It is important to note that compounds such as **19** and **21** reduced biofilm at the bottom of the well but was able to promote biofilm formation at the liquid–air interface, while an opposite trend was seen for compound **20**.

Among the biphenyl derivatives **11**, **13**, **14**, and **16**, only compound **14** possesses a methyl group at the *para* position on the aniline ring. It reduced both the total biofilm formation and the biofilm at the bottom of the well (64% reduction) (Figure 3B). The effect of the methyl group at the *para* position was better observed with the activities of **11** and **14**. While **14** appeared to be an inhibitor of the biofilm formed at the bottom of the well, **11** induced it by 14 times compared to the untreated control. Another finding worth noticing is that the change in 3-halogenic functionality from chloro (**11**) to bromo (**13**) reduced the biofilm formation at the bottom of the well (Figure 3B). Finally, by comparing **13** with **16**, a more polar compound appeared to have a stronger induction of biofilm formation at the bottom of the well (44% vs 735%; Figure 3B). The results also showed 5-alkylidenetetronamides as an antagonist against *E. coli* biofilm formation. For example, **20** reduced the total biofilm by 72%, although it only reduced the biofilm at the bottom of the well by 18%. In comparison, **21** was able to reduce the biofilm formation at the bottom of the well by 78% but had no significant reduction of the air–liquid interface biofilm.

Most of the other tetronamides enhanced biofilm formation. For example, tetronamides **6**–**9** showed an increased biofilm formation on the glass surface by 3–5 times compared to the control (Figure 3B). Interestingly, the transformation of **6** and **7** into the corresponding *syn*-aldol analogues **10** and **11** led to more biofilm formation. Compared to **7**, compound **11** increased *E. coli* biofilm formation at the bottom of the well by 3-fold and the total biofilms by 2.5-fold.

**Figure 4:**
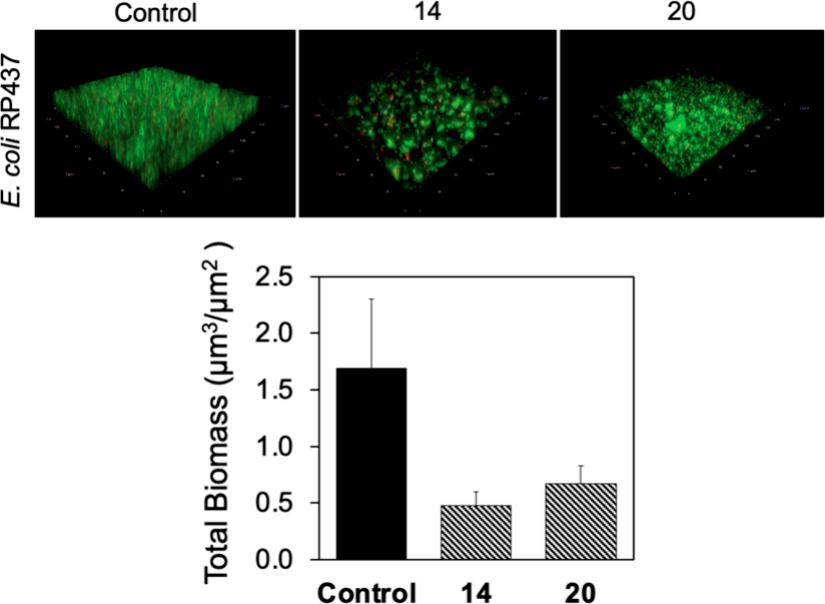
Representative fluorescence images of *E. coli* RP437 biofilms grown with (50 μg/mL) or without tetronamides. (Top) Biomass of *E. coli* RP437 biofilms grown with (50 μg/mL) or without tetronamides. (Bottom) Biomass was quantified from images using COMSTAT. The bar graph is the outcome of two experiments with five images taken from each sample.

Based on the total biofilm inhibition results, we selected compounds **14** and **20** to further corroborate the results using LIVE/DEAD staining to observe the biofilm structure under fluorescence microscopy. The results showed that 24 h *E. coli* RP437 biofilm formation was inhibited by biphenyl aldol **14** at 50 μg/mL (0.5 ± 0.1 μm^3^/μm^2^ vs 1.7 ± 0.6 μm^3^/μm^2^ of the untreated control) (Figure 4).

**Figure 5:**
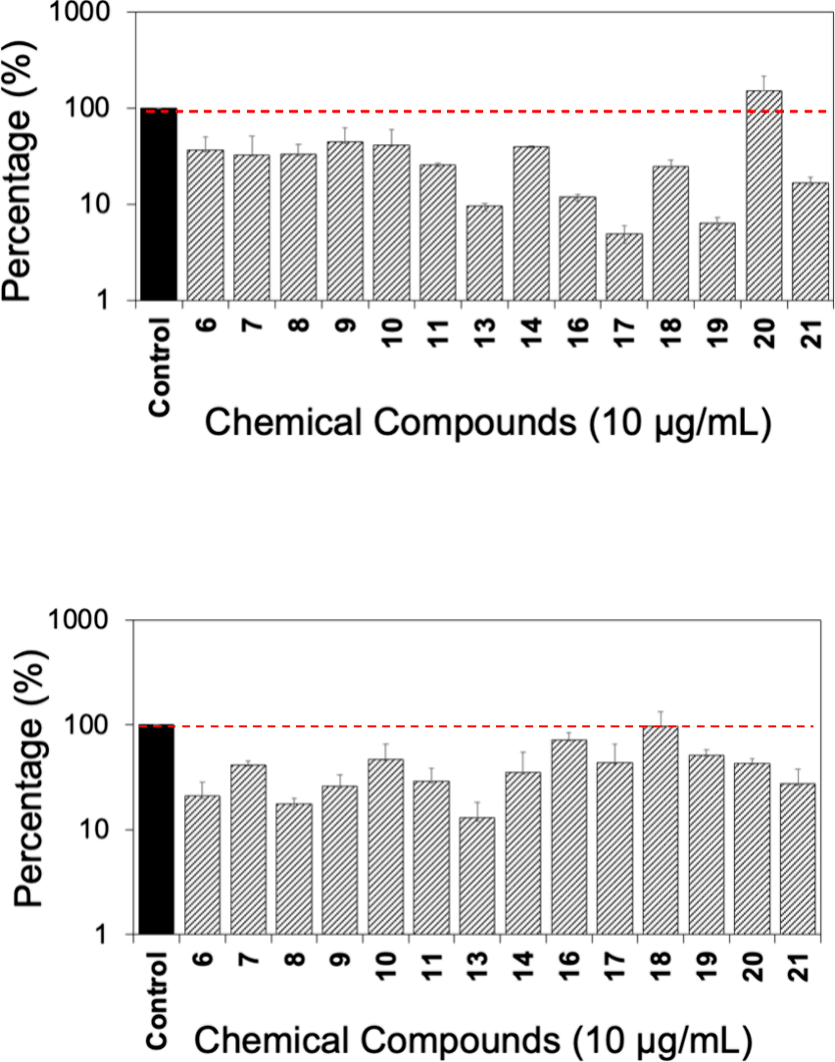
Effects of the tetronamides on QS. (A) AI-1 QS measured with the bioluminescence of reporter *V. harveyi* BB886 in response to the addition of candidate tetronamides (10 μg/mL). (B) AI-2 QS with the bioluminescence of *V. harveyi* BB170 in response to the addition of candidate tetronamides (10 μg/mL). Reporters without added tetronamides were used as controls. Means ± SE are presented (*n* = 2).

Since the compounds showed no killing of *E. coli* cells with the OD_600_ measurement, we then tested if biofilm reduction by effective compounds was through the interaction with QS. Although *E. coli* only has AI-2 QS,^54^ we tested the effects on QS via both AI-1 and AI-2 to obtain a full understanding of anti-QS activities. The effects of the compounds (10 μg/mL) on QS were investigated by employing the bioluminescence reporters *Vibrio harveyi* BB886 (AHL sensor + and AI-2 sensor −) and *V. harveyi* BB170 (AHL sensor – and AI-2 sensor +) (see Tables S1–S4).^55^ The results revealed that almost all tested compounds, to some degree, interfered with AI-1 and AI-2 QS, except for two inactive compounds: **20** (150%) for AI-1 and **18** (96%) for AI-2 (Figure 5A,B). These two compounds both have a methylenedioxy moiety attached to a benzene core. The most potent compounds interfering with AI-1 QS were **13** (91% reduction), **17** (95% reduction), and **19** (94% reduction) (Figure 5A). These compounds caused more than 10-fold reduction of QS measured based on the luminescence of the reporter *V. harveyi* BB886. Compound **16** (88% reduction) was also active against AI-1-mediated QS with slightly less activity than **13**. These results suggested a negative influence of the *para*-OH group on the aniline moiety. As noted, of the six biphenyl-aldolates (**11**–**16**), only **13** and **14** were among the most active species. The other two most active compounds (**17** and **19**) bear a phenyl moiety attached to electron-withdrawing groups (F and Cl). Significant inhibition of AI-2 QS was also observed, with **13** exhibiting the strongest effects (87%) (Figure 5B).

Despite the significant effects of compounds **13**, **16**, **17**, and **19** as inhibitors of AI-1 or AI-2, none of them caused a significant inhibition of biofilm formation of *E. coli* RP437. As we have discussed, the most powerful inhibitors of *E. coli* biofilms are **14** and 5-alkylidenefuranone **20**. These compounds caused only a moderate inhibition (0–68%) of AI-2-mediated QS. A lack of correlation between the inhibition of biofilm formation and the effects on AI-1 and AI-2 systems is not surprising since other mechanisms of biofilm inhibition could be contributing to the observed effects. Similar results have been reported for cadiolide analogues.^56^

### Antibiofilm Activity of Denigrins

**Figure 6:**
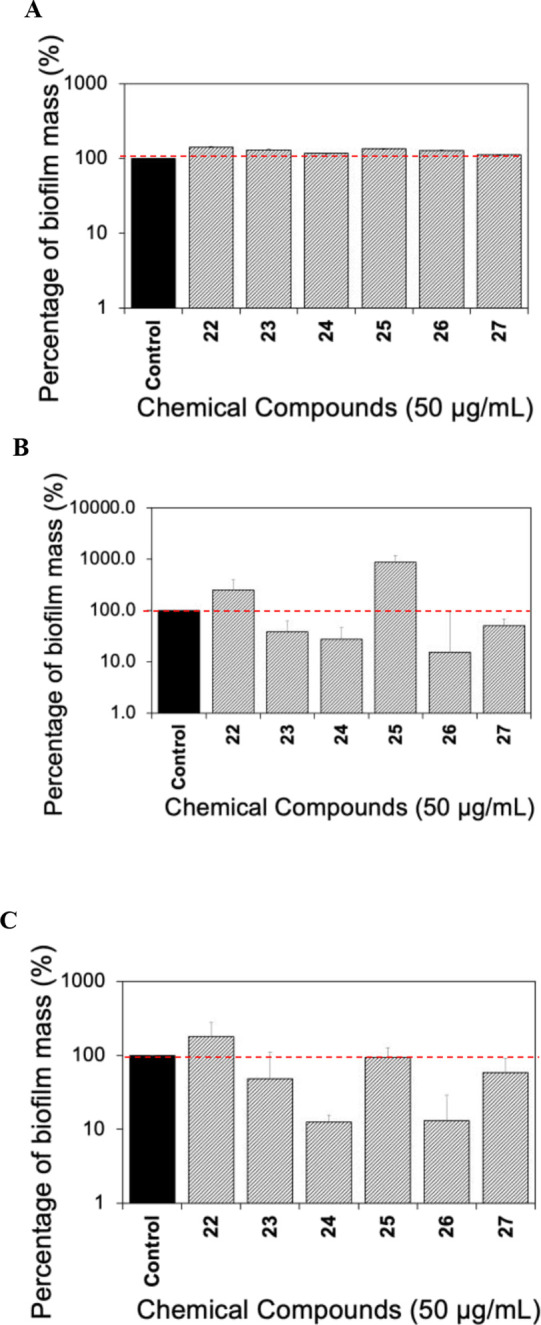
Crystal violet microtiter biofilm assay was used to evaluate the biofilm formation of *E. coli* RP437 when exposed to denigrins and precursors and compared to the untreated control. The results were normalized by corresponding cell-free media (either in LB or LB with 50 μg/mL of the compounds). (A) Total biofilm biomass based on OD_600_ measured to evaluate the effects of candidate compounds and normalized by the control as 100%. (B) Effects of the chemical compounds on bacterial biofilm formation at the bottom of the well. (C) Effects of the chemical compounds on total biofilm formation. Means ± SE are presented (*n* = 4).

As an inaugural investigation, natural denigrins and their synthetic intermediates were subjected to the same bioassays described for the tetronamide analogues. As observed in Figure 6A, in general, none of the compounds caused a major inhibition of bacterial growth when tested at 50 μg/mL.

In comparison, a significant inhibition of *E. coli* biofilm formation at the bottom of the well was observed for compounds **23** (by 62%), **24** (by 72%), **26** (by 85%), and **27** (by 49%) (Figure 6B). When considering the total biofilm inhibition of *E. coli*, compounds **24** (by 87%) and **26** (by 86%) were the most effective (Figure 6C). When comparing the effects of methylated compounds **24** and **26** with their demethylated analogues **25** (6%) and **27** (42%) (Figure 6C), it appears that an increase in polarity caused more total biofilm inhibition, similar to what was observed for tetronamide analogues.

In summary, the results in Figure 6 indicate that fully methylated analogues **24** and **26** are more effective against *E. coli* biofilm formation than **25** and **27**.

**Figure 7:**
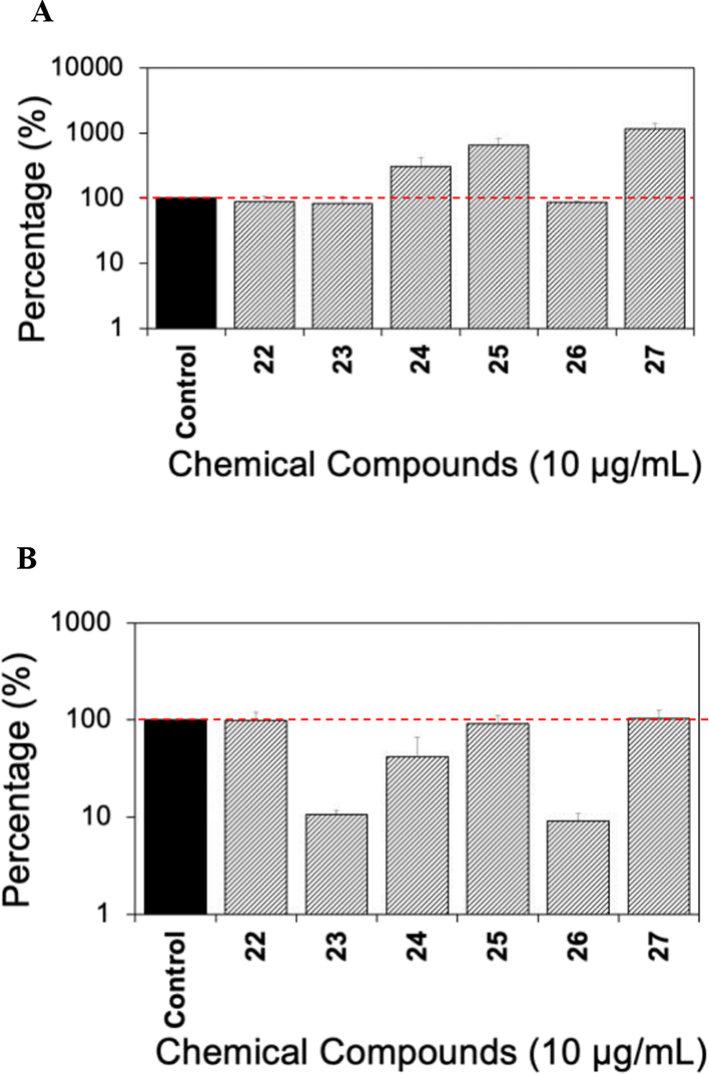
Effects of denigrins on QS. (A) Bioluminescence of *V. harveyi* BB886 in response to the addition of candidate denigrins and precursors (10 μg/mL) to measure AI-1 production. *V. harveyi* BB886 without added compound was used as the control. (B) Bioluminescence of *V. harveyi* BB170 in response to the addition of candidate denigrins and precursors (10 μg/mL) to measure AI-2 production. Means ± SE are presented (*n* = 2).

We investigated the effects of our synthesized compounds (at 10 μg/mL) on QS via AI-1 and AI-2 employing the same bioluminescence reporters as described above. The results in Figure 7 revealed that the compounds did not inhibit AI-1-mediated QS. On the other hand, compounds **23** (91% reduction) and **26** (91% reduction), possessing a *p*-methoxybenzylidene moiety, showed a 10-fold inhibition of QS via AI-2 compared to the control. It is important to point out that both compounds **23** and **26** were effective in inhibiting biofilm formation of *E. coli* (both at the bottom of the well and the total biofilm) (Figure 7). However, compound **24** that does not have a *p*-methoxybenzylidene moiety was very effective in inhibiting (by 91%) the total biofilm formation of *E. coli* but had no significant effect on AI-2 QS. These results indicate that the *p*-methoxybenzylidene moiety might play an important role in the inhibition of AI-2 QS, while the antibiofilm activity may be attributed to either a *p*-methoxybenzylidene or *p*-methoxyphenethyl hydrophobic moiety. However, for this class of compounds, factors other than QS may also be involved in biofilm inhibition, which deserves further study.

**Figure 8:**
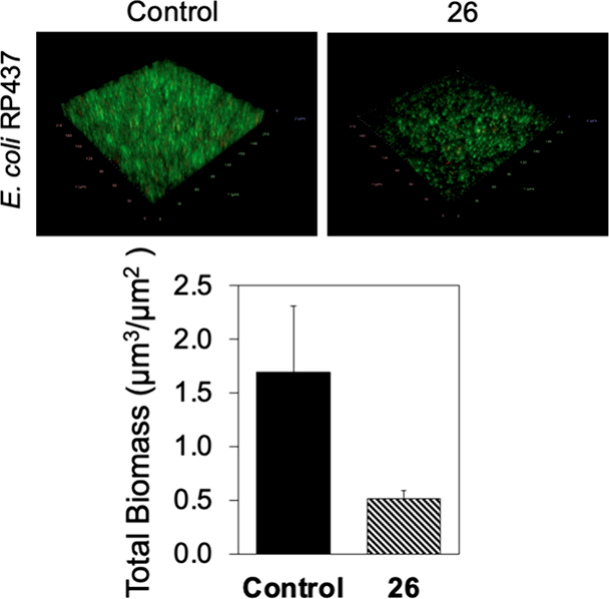
(Top) Representative fluorescence images of *E. coli* RP437 biofilms grown without (control) and with compound **26** (at 50 μg/mL). (Bottom) Quantified biomass from images after 24 h of biofilm grown with compound **26**. Control samples were grown in LB only. The bar graph is the outcome of two experiments with five images analyzed for each sample.

Given the potent effect on QS, the reduction of *E. coli* biofilm formation by compound **26** was further evaluated using imaging (Figure 8). The results show a significant reduction of biomass by compound **26** (0.5 ± 0.1 μm^3^/μm^2^ vs 1.7 ± 0.6 μm^3^/μm^2^ for the control).

**Figure 9:**
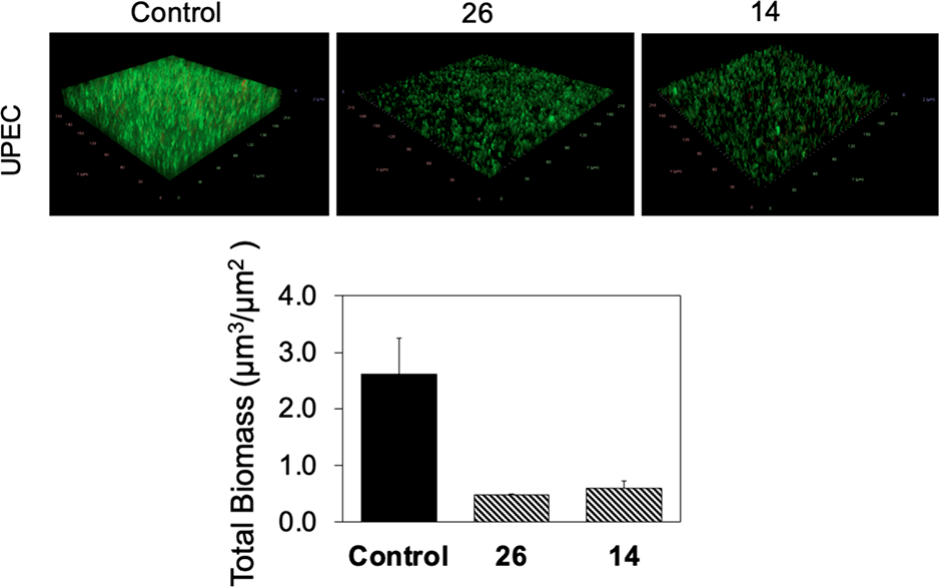
UPEC biofilms were grown for 24 h with 50 μg/mL compound **26** or **14** and labeled with LIVE/DEAD staining. (Top) Representative fluorescence images of UPEC biofilms grown with compound and compared with the control grown only in LB stained. (Bottom) Biomass of the images quantified using COMSTAT.

Compounds **14** and **26** showed potent activity in inhibiting the biofilm formation of *E. coli* RP437. To further test these two compounds on other pathogenic species, we compared their effects on 24 h biofilm formation of uropathogenic *E. coli* (UPEC). Urinary tract infections (UTIs) and catheter-associated UTIs (CAUTIs) are common healthcare-associated infections.^57^ Our results show a significant reduction of biomass by both compounds **14** (0.6 ± 0.1 μm^3^/μm^2^) and **26** (0.5 ± 0.0 μm^3^/μm^2^) when compared to the control (2.6 ± 0.6 μm^3^/μm^2^) (Figure 9). It is encouraging that similar effects were observed against both RP437 (a laboratory strain) and UPEC (a pathogen).

## Conclusions

In summary, we prepared a series of 14 tetronamides (including five new compounds) and six natural denigrins and their intermediates to investigate their effects on planktonic growth and biofilm formation of *E. coli*. In general, when tested at 50 μg/mL, most compounds had no significant effect on planktonic growth. The tetronamides had variable effects on the biofilm formation of *E. coli*, with the most active ones being **14** and **20**, causing 60 and 72% inhibition of total biofilm formation, respectively. The same series of tetronamides was also tested for their effects on AI-1 and AI-2 QS systems. In the QS assay using *V. harveyi* BB886, compounds **13**, **17**, and **19** caused at least a 10-fold reduction in the QS via AI-1. Compound **16** was also active against this target (AI-1), causing an 88% reduction, slightly less active than **13**. Despite the potent activity of some tested tetronamides, we found no clear correlation between the inhibition of *E. coli* biofilm formation and QS via AI-1 or AI-2. For denigrins, our results indicate that (1) an increase in polarity might be detrimental for the pursued bioactivity and (2) although either the *p*-methoxybenzylidene or *p*-methoxyphenetyl group might be essential for the antibiofilm activity (as indicated by the inactivity of **22** that lacks such a moiety), the former might be necessary for the inhibition of AI-2-mediated QS. Although a strong structure–activity relation was not found, several compounds showed promising activities against biofilm formation and/or QS. Further studies with other bacterial species and more tetronamides and denigrins can help further development of new compounds to better control bacterial infections.

## Materials and Methods

### Chemical Synthesis

For details on the experimental synthetic procedures and full spectroscopic data for new compounds, see the Supporting Information material. For details on the preparation and spectroscopic data for known compounds, see references in our previously published works.^46−50^

### Bacterial Strains and Culture Conditions

*E. coli* RP437 and uropathogenic *E. coli* (UPEC) ATCC53505 were grown overnight in lysogeny broth (LB) containing 10 g/L tryptone, 5 g/L yeast extract, and 10 g/L sodium chloride at 37 °C with constant shaking at 200 rpm. *V. harveyi* BB170 (AI-2) and *V. harveyi* BB886 (AHL) reporter strains were grown at 30 °C in autoinducer bioassay medium (AB) containing 17.5 g/L NaCl, 12.3 g/L MgSO_4_, and 2.0 g/L Casamino acids with pH adjusted to 7.5. Ten milliliters of 1 M KH_2_PO_4_ (pH 7.0), 10 mL of 0.1 M l-arginine, 10 mL of glycerol, 1 mL of 10 μg/mL riboflavin, and 1 mL of 1 mg/mL thiamine were added after sterilization. l-Marine (LM) plates containing 10 g/L tryptone, 5 g/L yeast extract, 20 g/L NaCl, and 15 g/L agar were used to count the number of colonies after incubation overnight at 30 °C.

### Biofilm Assay

A microtiter plate-based crystal violet assay was used to evaluate the biofilm formation. Crystal violet is a basic dye that can bind to negatively charged surface molecules including polysaccharides and thus stain cells in purple color. Each chemical compound was dissolved in DMSO. Some chemical compounds were exposed to heat (60 °C) first to fully dissolve. *E. coli* RP437 was grown in sterile flat bottom 96-well plates. Each well was inoculated with an initial OD_600_ of 0.05 in a total volume of 300 μL of the LB medium. Each compound was tested at 50 μg/mL. The plates were incubated for 24 h at 37 °C without shaking. An initial growth reading was taken to record the total growth at OD_600_. Afterward, the medium with planktonic cells was aspirated and washed three times with sterile DI water. The plates were left to dry for 5 min. Then, 300 μL of crystal violet solution (0.1% in water) was added to each well and incubated for 20 min at room temperature to dissolve the bound crystal violet. Then, the plates were washed three times with sterile DI water. A reading of OD_590_ was taken to measure the liquid–solid interface biofilms at the bottom of each well (static measurement). Then 95% ethanol was added to each well and shaken for 30 s. A reading was then taken at OD_590_ to quantify total biofilms including those at the air–liquid interface. The readings were calibrated by subtracting the cell-free background signals.

### QS Assay

Bioassays were performed with *V. harveyi* BB170 (AI-2) and *V. harveyi* BB886 (AI-1) reporter strains grown at 30 °C in autoinducer bioassay medium, which contains 17.5 g/L NaCl, 12.3 g/L MgSO_4_, and 2.0 g/L Casamino acids with pH adjusted to 7.5 and the addition of 10 mL of 1 M KH_2_PO_4_ (pH 7.0), 10 mL of 0.1 M l-arginine, 10 mL of glycerol, 1 mL of 10 μg/mL riboflavin, and 1 mL of 1 mg/mL thiamine. Cell-free supernatants were prepared by taking overnight cultures of the reporter strains and centrifuged at 13,200 rpm for 10 min at 4 °C. The supernatant was then sterilized by filtering through a 0.22 μm membrane filter. The cell-free supernatants were stored at −20 °C until use. Overnight cultures of *V. harveyi* BB170 and *V. harveyi* BB886 were grown in autoinducer bioassay medium (AB) and diluted 1:5000 into fresh AB medium. Cell-free supernatants (10% v/v) were added to measure AI-1 or AI-2 activity and compared to samples without cell-free supernatants in a 96-well plate. Relative bioluminescence of growing *V. harveyi* cultures was measured every hour using a Turner Designs 20/20 luminometer. Cell density was measured by diluting the same sample that was used for light production and plating it on l-marine plates containing 10 g/L tryptone, 5 g/L yeast extract, 20 g/L NaCl, and 15 g/L agar. The number of colonies was counted after growth overnight at 30 °C. To identify which compounds can inhibit either AI-1 or AI-2 QS, an appropriate *V. harveyi* reporter with an added supernatant was grown in a 96-well plate. At a predetermined time point (3 h for BB886 and 5.5 h for BB170), each compound was added at a concentration of 10 μg/mL and the bioluminescence and cell density were measured after 1.5 h of incubation. Each compound was tested in duplicate.

### Biofilm Formation and Imaging

Biofilms were grown on glass slides. Glass slides (7.0 mm × 10.0 mm; 1 mm thickness) were sterilized with 100% ethanol and dried in a 50 °C oven overnight. The biofilms were inoculated with a starting OD_600_ of 0.05 using overnight culture on glass slides with 20 mL of LB. The biofilms were grown for 24 h without shaking. After 24 h, the biofilms were gently washed three times with 0.85% NaCl solution and stained with LIVE/DEAD BacLight Bacterial Viability Kit (Life Technologies Inc., Carlsbad, CA, USA) in 3 mL of PBS solution supplemented with 4.5 μL of each component, SYTO9 and propidium iodide (PI). Fluorescence images of biofilms were taken using an Axio Imager M1 fluorescence microscope (Carl Zeiss Inc., Berlin, Germany) and analyzed using COMSTAT.^58^

## Supporting Information Available

Characterization data of newly synthesized compounds and copies of the ^1^H and ^13^C NMR spectra (PDF)
