# Supplementary material for: Effects of Synthetic Tetronamides and Methylated Denigrins on Bacterial Quorum Sensing and Biofilm Formation
Source: ACS Omega. 2023 Oct 2;8(41):37798–807. doi: 10.1021/acsomega.3c01729 (PMC10586261; doi:10.1021/acsomega.3c01729)
Supplement: Supplementary file 1 — ao3c01729_si_001.pdf [file ao3c01729_si_001.pdf]

SUPPORTING INFORMATION

Effects of Synthetic Tetronamides and Methylated Denigrins on Bacterial Quorum Sensing and Biofilm Formation

Sweta Roy[1,†] Jaime A. M. Acosta,[2,3,†] Milandip Karak,[2,4,†] Isabela Ramirez-Velez,[1] Kohei Torikai,[4,5,]* Dacheng Ren,[1,6,7,]* and Luiz C. A. Barbosa[2,]*

[1]Department of Biomedical and Chemical Engineering, Syracuse University, Syracuse, NY 13244, United States
[2]Department of Chemistry, Universidade Federal de Minas Gerais, Av. Pres. Antônio Carlos, 6627, Campus Pampulha, CEP 31270-901, Belo Horizonte, MG, Brazil
[3]Chemical Technology School, Universidad Tecnológica de Pereira, Carrera 27 #10-02, Barrio Alamos, Código postal: 660003, Pereira, Risaralda, Colombia
[4]Department of Chemistry, Faculty of Science, Kyushu University, 744 Motooka, Nishi-ku, Fukuoka 819-0395, Japan
[5]Faculty of Chemistry, National University of Uzbekistan named after Mirzo Ulugbek, 4 University Str., Tashkent 100174, Uzbekistan
[6]Department of Civil and Environmental Engineering, Syracuse University, Syracuse, NY 13244, United States
[7]Department of Biology, Syracuse University, Syracuse, NY 13244, United States

† **S.R.**, **J.A.M.A.**, and **M.K.** Contributed equally.
Correspondence to: **K.T.** (torikai@chem.kyushu-univ.jp); **D.R.** (dren@syr.edu); **L.C.A.B.** (lcab@ufmg.br)

1. General information

To perform all reactions, analytical grade solvents were used without further purifications, unless otherwise stated. The ^{1}H and ^{13}C NMR spectra were recorded on a Varian Mercury 300 instrument (300 MHz and 75 MHz, respectively) or on a Bruker NMR spectrometer (400 MHz and 100 MHz, respectively). The samples were dissolved in deuterated chloroform, acetone or dimethyl sulfoxide (DMSO), and tetramethylsilane (TMS) was used as internal standard (δ = 0). The experiments were performed at controlled probe temperature of 25 °C. Chemical shifts of ^{1}H and ^{13}C NMR spectra are reported in ppm. All coupling constants (*J* values) are expressed in Hertz (Hz). Multiplicities are reported as follows: singlet (s), doublet (d), doublet of doublets (dd), triplet (t), multiplet (m) and broad (br). High resolution mass spectra were recorded on a Bruker MicroTof (resolution = 10000 FWHM) under electrospray ionization. The reactions were monitored by analytical thin layer chromatography analysis performed on aluminum packed pre-coated silica gel plates. All compounds were purified by column chromatography using silica gel (230–400 mesh) as a solid stationary phase.

2. Chemical synthesis

The starting tetronamides of the present work were prepared following a procedure that we previously reported.[1] The desired 3-halotetronamides **6-9, S3-S5,** and **S7** were prepared from commercially available 3,4-dihalofuran-2(5*H*)-ones **S1** and **S2** with pyrrolidine or aromatic amines in the presence of sodium bicarbonate at room temperature *via* an *aza*-Michael addition/elimination reaction sequence (Figure S1, Step-1). In addition to 3-halotetronamides, 3-dehalogenated compound **S6** was obtained in high yield from 4-amino-3-halosubstituted butenolides **S4-S5** by following a reductive hydrodehalogenation protocol with the combination of a palladium catalyst and formic acid/DIPEA as a hydrogen source.[2] Next, compounds **10-15, S8** and **17-19** were obtained by the direct vinylogous aldol reaction between appropriate aromatic/heteroaromatic aldehydes and parent tetronamides **6-9, S3-S7** by using sodium hydroxide or lithium hydroxide monohydrate in methanol : water (2:1, v/v) at room temperature (Figure S1, Step-2).[1] In most cases the *syn* aldol adducts were formed as major isomers, in agreement with our previous observations.[1, 3] Detailed discussion and the synthetic procedure to prepare such tetronamide aldolates have already been reported, along with the structural characterizations of the known compounds.[1, 3, 4]

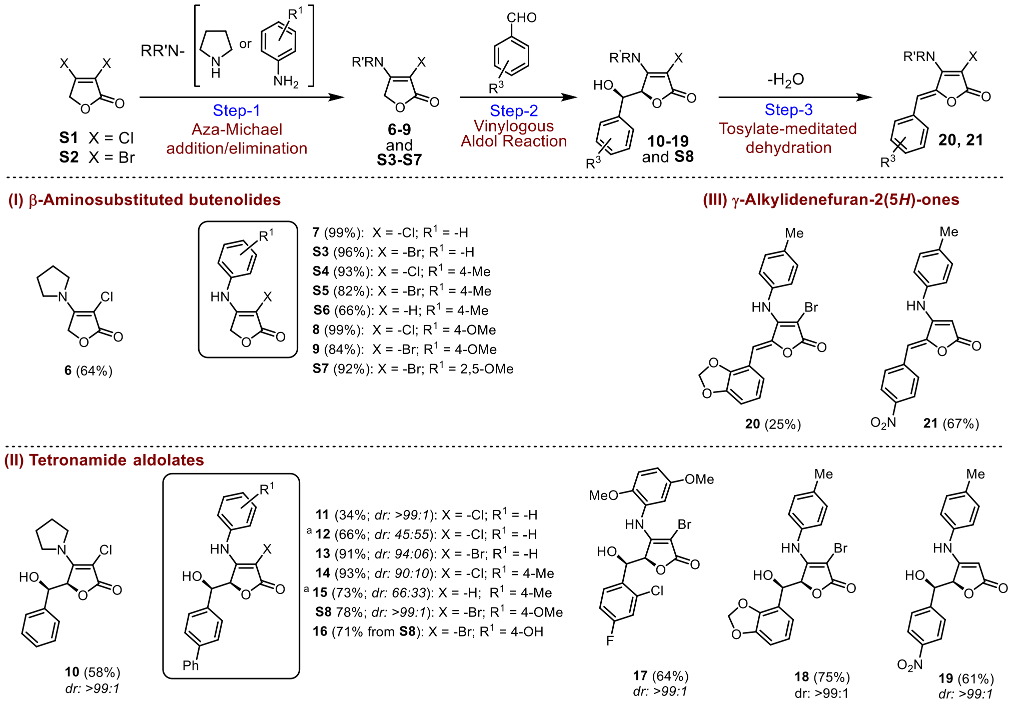

Figure S1. General procedure of synthesis to prepare tetronamide derivatives. For the purpose of clarity only the *syn* isomers are shown along with the *syn:anti* ratio. Unless indicated otherwise, pure *syn*-isomers of tetronamide aldolates are tested for antibiofilm activities. [a]Compounds **12** and **15** are non-separable mixture of diastereomers and tested for antibiofilm activities *as is*.

Furthermore, treatment of compound **S8** with boron tribromide in dichloromethane accomplished removal of the methyl group to afford phenolic compound **16** in high yield. Finally, compounds **18** and **19** were treated with 4-toluenesulfonyl chloride in the presence of 1,8-diazabicyclo[5.4.0]undec-7-ene and acetonitrile to afford the desired (*Z*)-5-benzylidenetetronamides **20** and **21** in good yields (Figure S1, Step-3), respectively.[4]

2.1. Synthetic procedures and characterization data

*Procedure for the preparation of compounds **6-9** and **S3-S7***

Compounds **7-9** and **S3-S6** were previously reported by our group and the detailed synthetic procedure, physical and spectroscopic data can be found from the respective references.[1, 3, 4] Compounds **6** and **S7** were synthesized using a method similar to that described for compounds **7-9** and **S3-S6**. The physical and spectroscopic data for **6** and **S7** are shown below.

3-chloro-4-(pyrrolidin-1-yl)furan-2(5H)-one **(*6*)**

The crude residue was purified by silica gel column chromatography eluting with hexane/ethyl acetate (1:1 v/v) to afford compound **6** as a white solid in 64% yield. M.p. 126.7-128.2 ºC. ^{1}H NMR (400 MHz, $CDCl_3$) δ 4.64 (s; 2H; H-5), 3.88 (brd; 2H, H-1' and H-4'), 3.33 (brd; 2H, H-1' and H-4'), 1.98 (s; 4H; H-2' and H-3'). ^{13}C NMR (100 MHz, $CDCl_3$) δ 171.12 (C-2), 157.02 (C-4), 91.54 (C-5), 84.33 (C-3), 66.26 (2C, C-1' and C-4'), 48.58 (2C, C-2' and C-3'). HRMS (ESI) *m/z* $[M+Na]^+$: calculated for $C_8H_{10}ClNNaO_2$: 210.0293, found: 210.0289.

3-chloro-4-((2,5-dimethoxyphenyl)amino)furan-2(5H)-one ***(S7)***

The crude residue was purified by silica gel column chromatography eluting with hexane/ethyl acetate (1:1 v/v) to afford compound **S7** as a white solid in 92% yield. M.p. 142.5 -143.8ºC. ^{1}H NMR (400 MHz, $CDCl_3$) δ 7.22 (s, 1H, -NH), 6.87 (d, *J* = 8.9 Hz, 1H, H-3′), 6.63 (dd, *J* = 8.8 and 2.2 Hz, 1H, H-4'), 6.47 (d, *J* = 2.3 Hz, 1H, H-6'), 5.03 (s, 2H, H-5), 3.86 (s, 3H, H-8′) and 3.78 (s, 3H, H-7′). ^{13}C NMR (100 MHz, $CDCl_3$) δ 169.12 (C-2), 159.37 (C-5'), 154.05 (C-4), 144.23 (C-2') 127.83 (C-1'), 112.13 (C-3'), 108.55 (C-4'), 106.45 (C-6'), 79.23 (C-3), 67.60 (C-5), 56.50 (C-8') and 55.93 (C-7′). HRMS (ESI) calculated for $C_{12}H_{11}BrNO_4^-$ $[M-H]^-$, 311.9877; found, 311.9873.

*Procedure for the preparation of compounds **10-19** and **S8***

Compounds **11-15, S8, 16,** and **19** were previously reported by our group and the detailed synthetic procedure, spectroscopic and physical data can be found from the respective references.[1, 3, 4] However, most of these compounds were re-prepared for use in this study and all characteristic data are in agreement with our previous report. Compounds **10**, **17**, and **18** were synthesized using a method similar to that described for compounds **11-15, S8, 16,** and **19**. The physical and spectroscopic data for new compounds **10**, **17**, and **19** are shown below.

3-chloro-5-(hydroxy(phenyl)methyl)-4-(pyrrolidin-1-yl)furan-2(5H)-one ***(10)***

The crude residue was purified by silica gel column chromatography eluting with hexane/ethyl acetate (35:65 v/v) to afford compound **10** as a white solid in 58% yield. M.p. 225.4-225.9 ºC. ^{1}H NMR (400 MHz, $(CD_3)_2SO$) δ 7.44 (d, *J* = 7.5 Hz, 2H, H-2'' and H-6''), 7.36 (t, *J* = 7.3 Hz, 2H, H-3'' and H-5''), 7.44 (d, *J* = 7.1 Hz, 1H, H-4''), 5.72 (d, *J* = 5.5 Hz, 1H, H-5), 5.32 (s, 1H, -OH), 5.12 (d, *J* = 5.6 Hz, 1H, H-6), 5.73 (brd, 2H, H-1' and H-4'), 5.72 (brd, 2H, H-1' and H-4'), 5.13 (brd, 2H, H-2' and H-3'), 5.12 (brd, 2H, H-2' and H-3'). DEPT135 NMR (100 MHz, $(CD_3)_2SO$) δ 169.39 (C-2), 158.03 (C-4), 141.27 (C-1'), 127.77 (2C, C-3'' and C-5''), 127.17 (C-4''), 126.42 (2C, C-2'' and C-6''), 84.15 (C-3), 80.61 (C-5), 69.03 (C-6), 40.36 (2C, C-1' and C-4'), 24.81 (2C, C-2' and C-3'). HRMS (ESI) *m/z* $[M+Na^+]^+$: calculated for $C_{15}H_{16}ClNNaO_3$, 316.0711; found, 316.0710.

3-chloro-4-((2,5-dimethoxyphenyl)amino)furan-2(5H)-one ***(17)***

The crude residue was purified by silica gel column chromatography eluting with hexane/ethyl acetate (20:80 v/v) to afford compound **17** as a white solid in 64% yield. M.p. 268.7-269.2 ºC. ^{1}H NMR (400 MHz, DMSO-d_6) δ 9.14 (s, 1H, -NH), 7.56 (t, *J* = 7.6 Hz, 1H, H-5''), 7.30-7.22 (m, 2H, H-6'' and H-3''), 7.06 (d, *J* = 8.9 Hz, 1H, H-3'), 6.94 (s, 1H, H-6'), 6.88 (d, *J* = 8.9 Hz, 1H, H-4'), 5.97 (d, *J* = 4.6 Hz, 1H, H-5), 5.11 (s, 1H, -OH), 4.84 (d, *J* = 4.6 Hz, 1H, H-6), 3.77 (s, 1H, H-8') and 3.72 (s, 3H, H-7'). ^{13}C NMR (100 MHz, DMSO-d_6) δ 169.05 (C-2), 161.93 (C-4), 161.13 (d, *J* = 247.1 Hz, C-4''), 153.13 (C-5'), 147.86 (C-2'), 134.72 (d, *J* = 3.0 Hz, C-1''), 131.12 (d, *J* = 8.9 Hz, C-2''), 130.47 (d, *J* = 10.7 Hz, C-6''), 126.78 (C-

1'), 115.70 (d, J = 25.1 Hz, C-3''), 114.11 (d, J = 21.4 Hz, C-5''), 113.76 (C-3'), 113.32 (C-6'), 112.58 (C-4'), 79.34 (C-3), 75.46 (C-5), 65.63 (C-6), 56.05 (C-7'), 55.54 (C-8′). HRMS (ESI) [M−H]$^-$ calculated for $C_{19}H_{15}Cl_2FNO_5$, 469.9812; found, 469.9809.

(R)-5-((R)-benzo[d][1,3]dioxol-5-yl(hydroxy)methyl)-3-bromo-4-(p-tolylamino)furan-2(5H)-one ***(18)***
The crude residue was purified by silica gel column chromatography eluting with hexane/ethyl acetate (35:65 v/v) to afford compound **18** as a white solid in 75% yield. M.p. 167.9-168.8 ºC. ^{1}H NMR (400 MHz, DMSO-d$_6$) δ 9.43 (s, 1H, -NH), 7.21 (s, 2H, H-5′ and H-3'), 7.19 (s, 2H, H-6′ and H-2'), 6.87 (d, J = 8.0 Hz, 1H, H-2''), 6.83 (s, 1H, H-6'), 6.71 (d, J = 8.0 Hz, 1H, H-5''), 5.99 (s, 2H, H-7''), 5.75 (s, 1H, H-6), 5.37 (s, 1H, H-5), 4.11 (s, 1H, -OH) and 2.31 (s, 3H, H-7'). ^{13}C NMR (100 MHz, DMSO-d$_6$) δ 169.6 (C-2), 160.0 (C-4), 147.0 (C-3''), 146.3 (C-4''), 135.4 (C-1'), 135.1 (C-4'), 134.6 (C-1''), 129.21 (2C, C-3' and C-5'), 124.1 (2C, C-2' and C-6'), 119.2 (C-5''), 107.7 (C-2''), 106.74 (C-6''), 81.8 (C-7''), 75.1 (C-5), 69.1 (C-6), 20.5 (C-7'). HRMS (ESI) calculated for $C_{19}H_{15}BrNO_5$ [M−H]$^-$, 416.0139; found, 416.0150.

Procedure for the preparation of compounds ***20-21***
Compound **21** was previously reported by our group and the detailed synthetic procedure, spectroscopic and physical data can be found from the respective reference.[4] Compound **20** was synthesized using a method similar to that described for compound **21**. The physical and spectroscopic data for **20** are shown below.

(Z)-5-(benzo[d][1,3]dioxol-5-ylmethylene)-3-bromo-4-(p-tolylamino)furan-2(5H)-one ***(20)***
The crude residue was purified by silica gel column chromatography eluting with hexane/ethyl acetate (85:15 v/v) to afford compound **20** as a white solid in 25% yield. M.p. 157.2-159.3 ºC. ^{1}H NMR (400 MHz, DMSO-d$_6$) δ 9.58 (s, 1H, -NH), 7.30 (s, 1H, H-6′), 7.24-7.12 (m, 3H, H-3', H-5' and H-2'), 7.10-7.00 (m, 3H, H-2', H-6' and H-6), 6.09 (s, 2H, H-7''), 2.31 (s, 3H, H-7'). HRMS (ESI) calculated for $C_{19}H_{13}BrNO_4$ [M−H]$^-$, 398.0033; found, 398.0029.

Denigrins A and B (**25** and **27**) were prepared from maleic anhydride in three and five steps in 62% and 31% overall yields, respectively (Figure S2).[5] The reaction of the maleic anhydride and diaryliodonium tetrafluoroborate, catalyzed by Pd(OAc)$_2$/NaOAc in acetonitrile provided 69% yield of diarylated

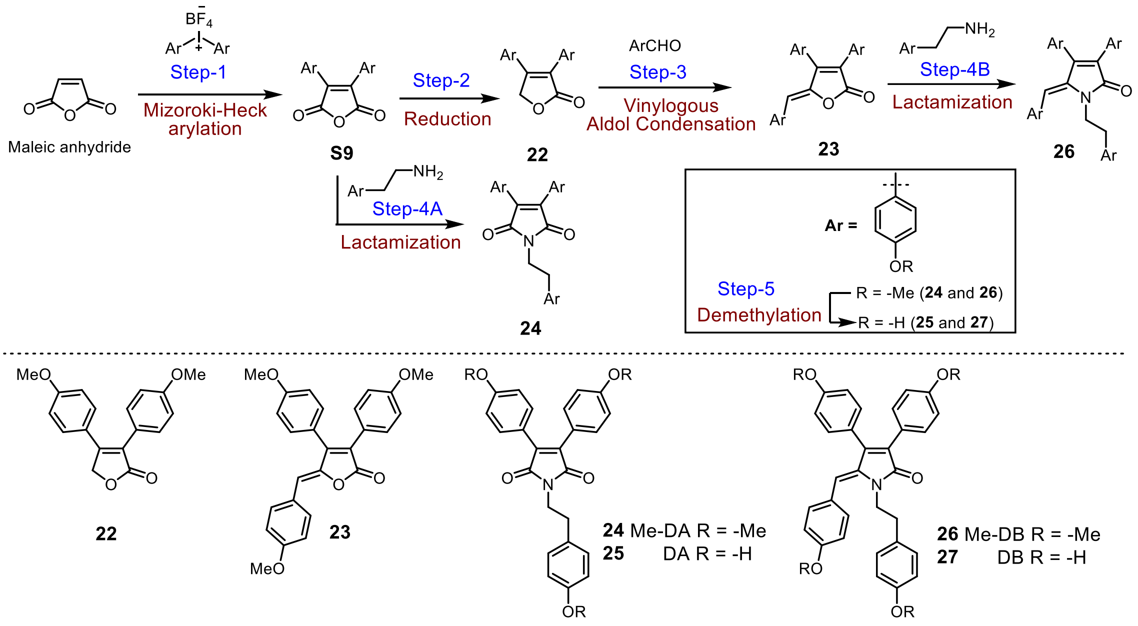

Figure S2. General synthetic procedure for denigrins A (**25**) and B (**27**).

compound **S9** (Step 1). With diarylated precursor in hands, a direct one-pot reaction of compound **S9** with 4-methoxyphenethylamine in acetic acid for 6 h (Figure S2, Step-4A) delivered methylated denigrin A **24** in 93% yield. On the other hand, same anhydride precursor **S9** was reduced with lithium aluminum hydride (Figure S2, Step-2) to afford butenolide motif **22** in 86% yield. Next, the vinylogous aldol condensation

(Figure S2, Step-3) of **22** with 4-methoxybenzaldehyde using sodium carbonate in MeOH furnished the desired *Z*-isomer of **23** as a sole product in 89% yield. Then the one-pot lactamization of compound **23** (Figure S2, Step-4B) afforded the methylated denigrin B **26** in 66% yield. As a final step, borontribromide-meditated demethylation (Figure S2, Step-5) yielded the natural denigrins A (**25**) and B (**27**) in excellent yields from **24** and **26**, respectively. The characterization of all compounds was carried out by spectroscopic analyses and are in agreement with previously reported data.[5]

*Procedure for the preparation of compounds **S9**, and **22-27***

Compounds **S9**, and **22-27** were previously reported by our group and the detailed synthetic procedure, spectroscopic and physical data can be found from the respective reference.[5]

3. Spectroscopic data for tetronamides 6 and S7

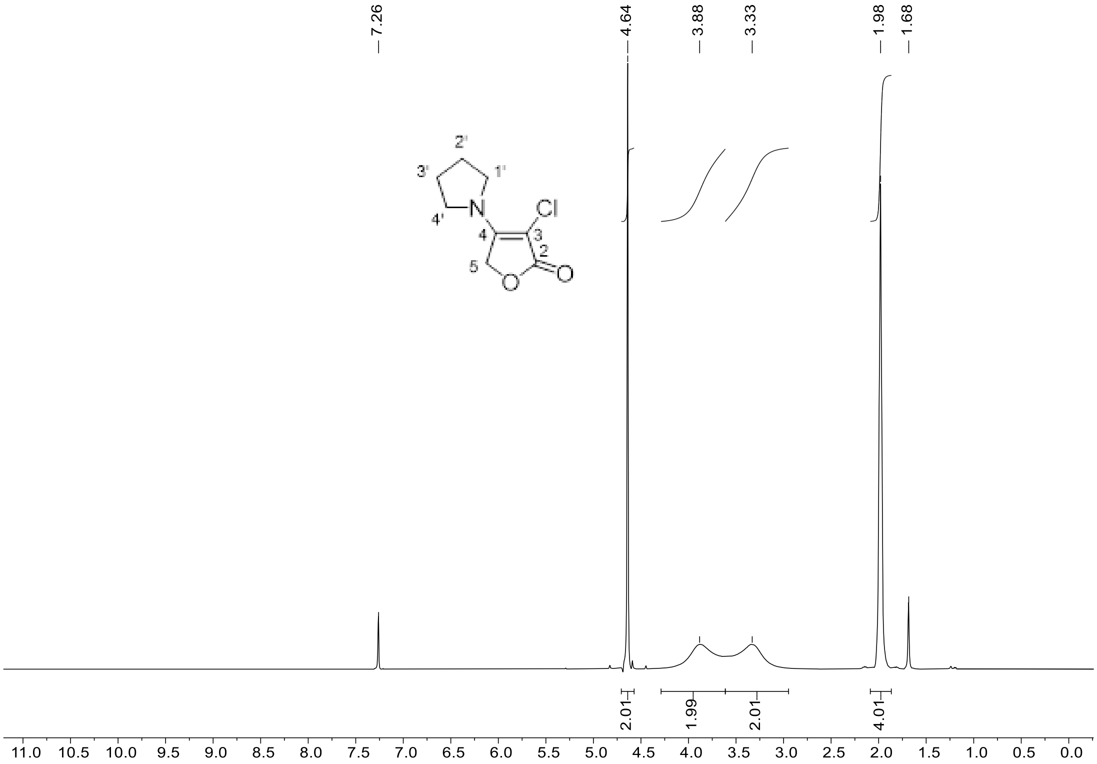

Figure S3: ^{1}H NMR (400 MHz, $CDCl_3$) of compound **6**.

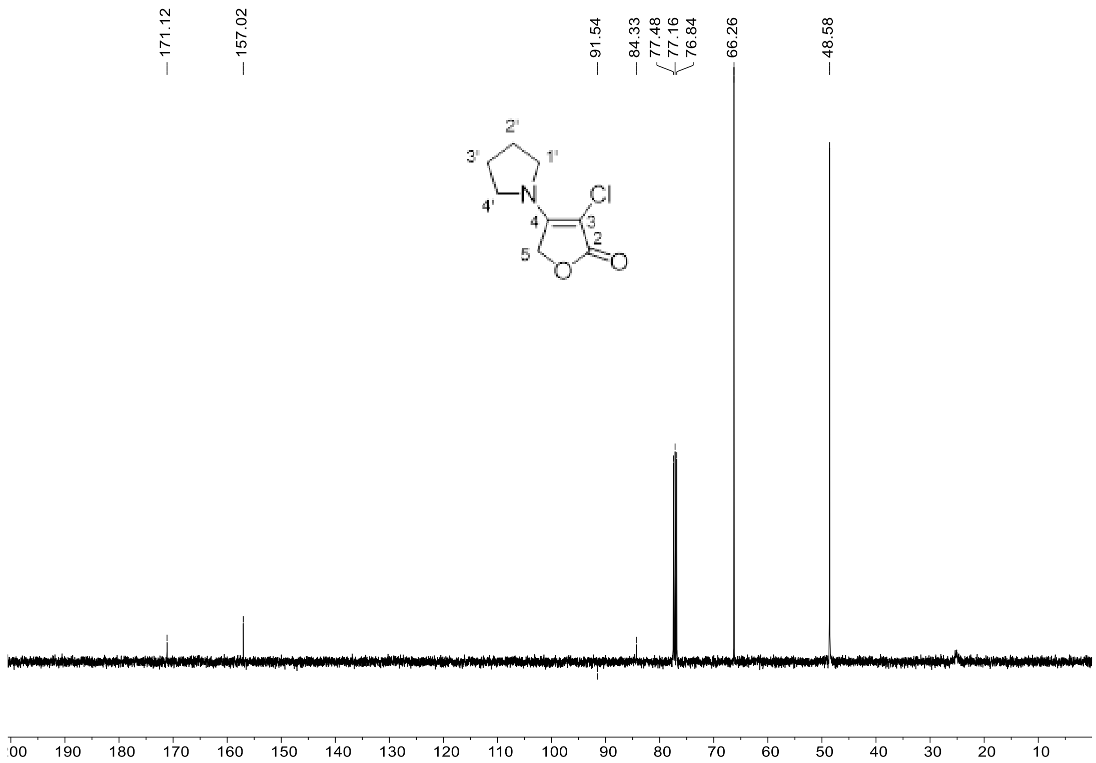

Figure S4: DEPT135 (100 MHz, $CDCl_3$) of compound **6**.

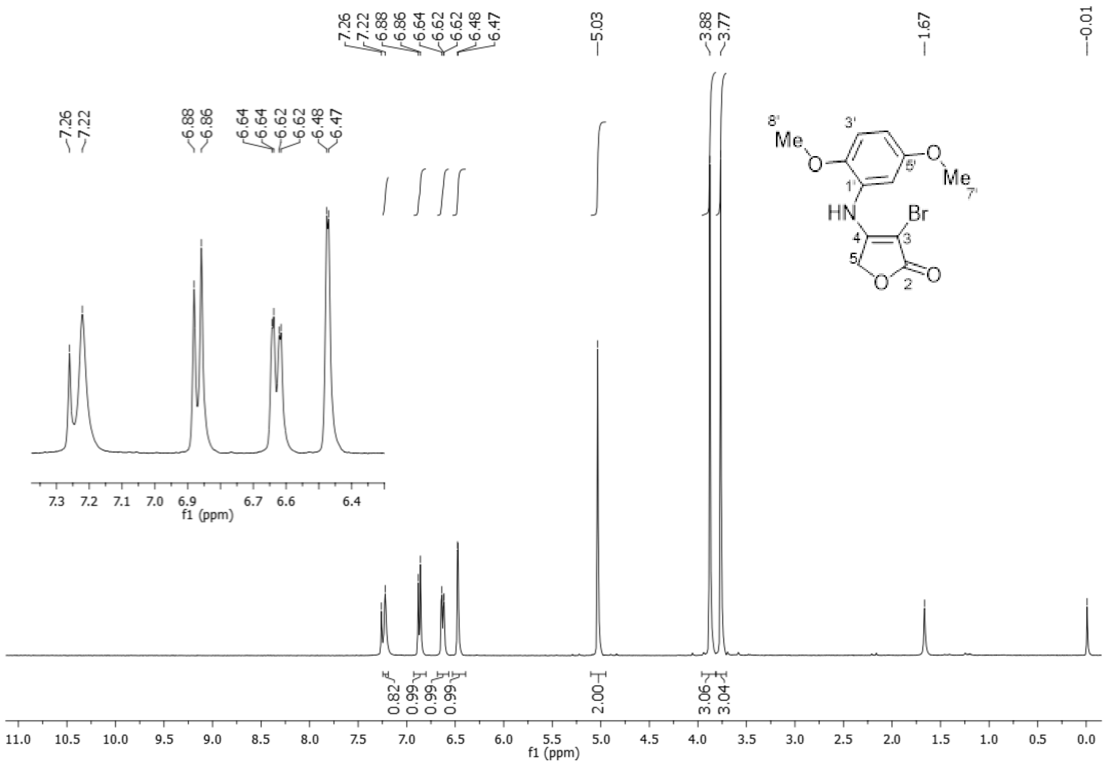

Figure S5: ^{1}H NMR (400 MHz, $CDCl_3$) of compound **S7**.

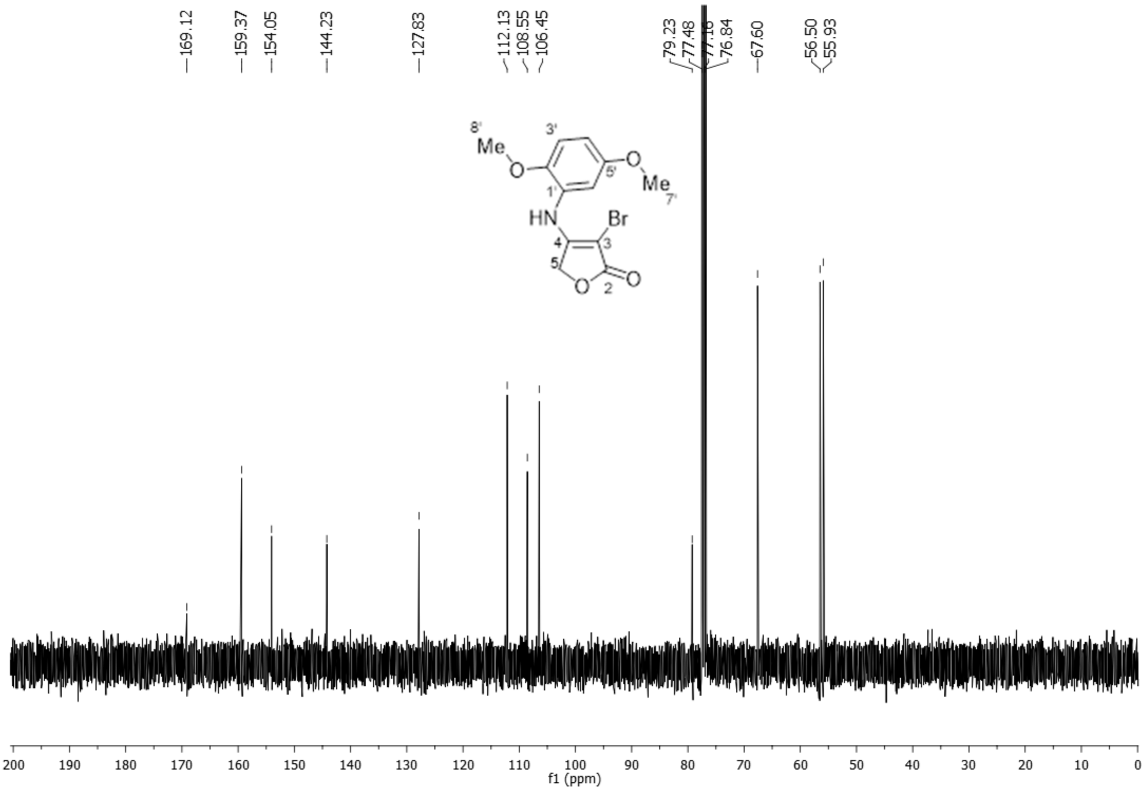

Figure S6: ^{13}C NMR (100 MHz, $CDCl_3$) of compound **S7.**

4. Spectroscopic data for tetronamide aldolates 10, 17 and 18

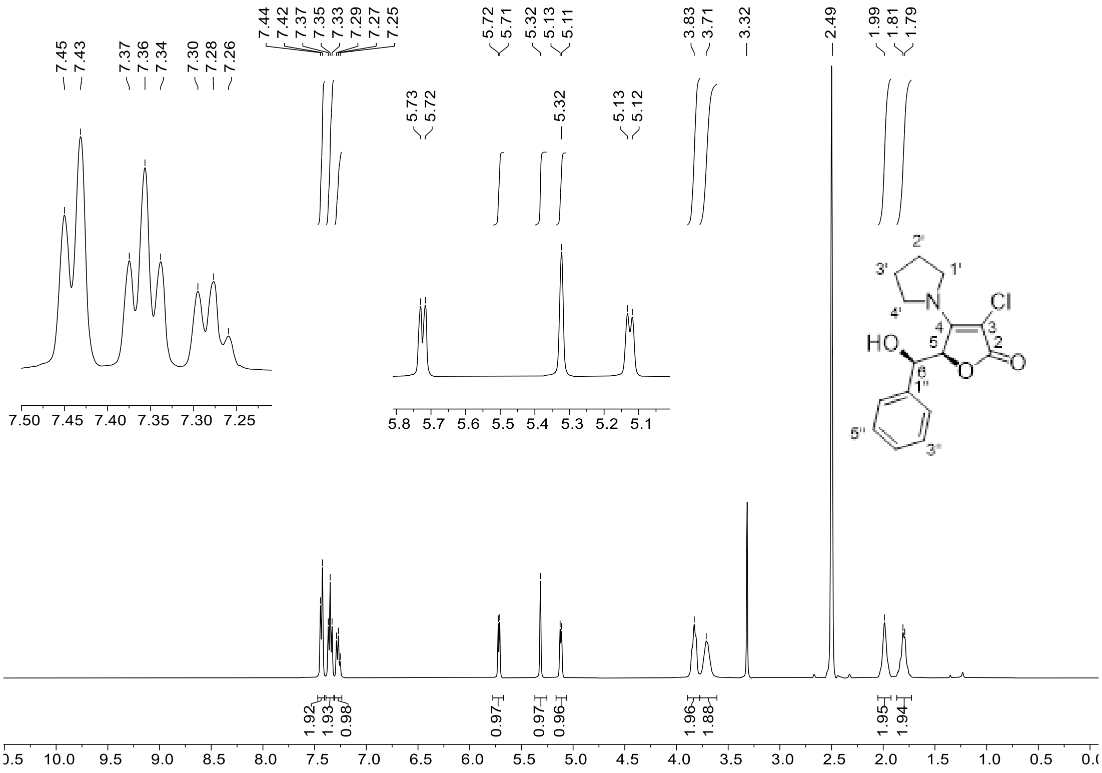

Figure S7: ^{1}H NMR (400 MHz; $(CD_3)_2SO$) of compound **10**.

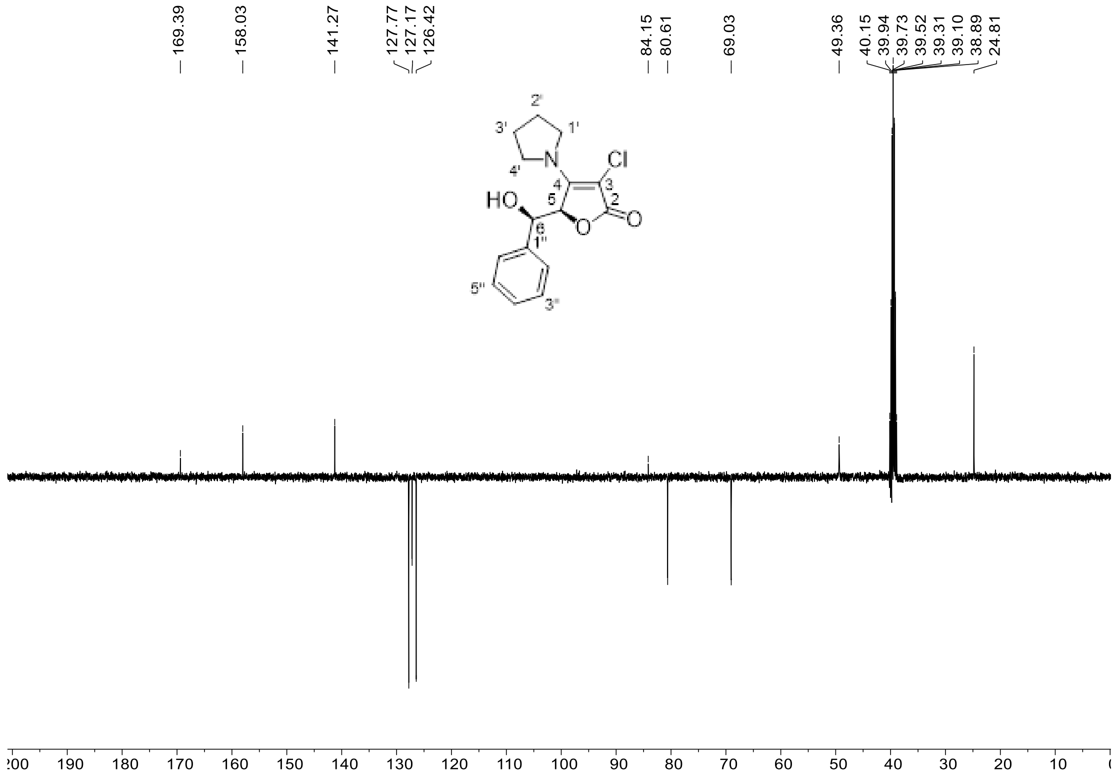

Figure S8: DEPT135 NMR (100 MHz, $(CD_3)_2SO$) of compound **10**.

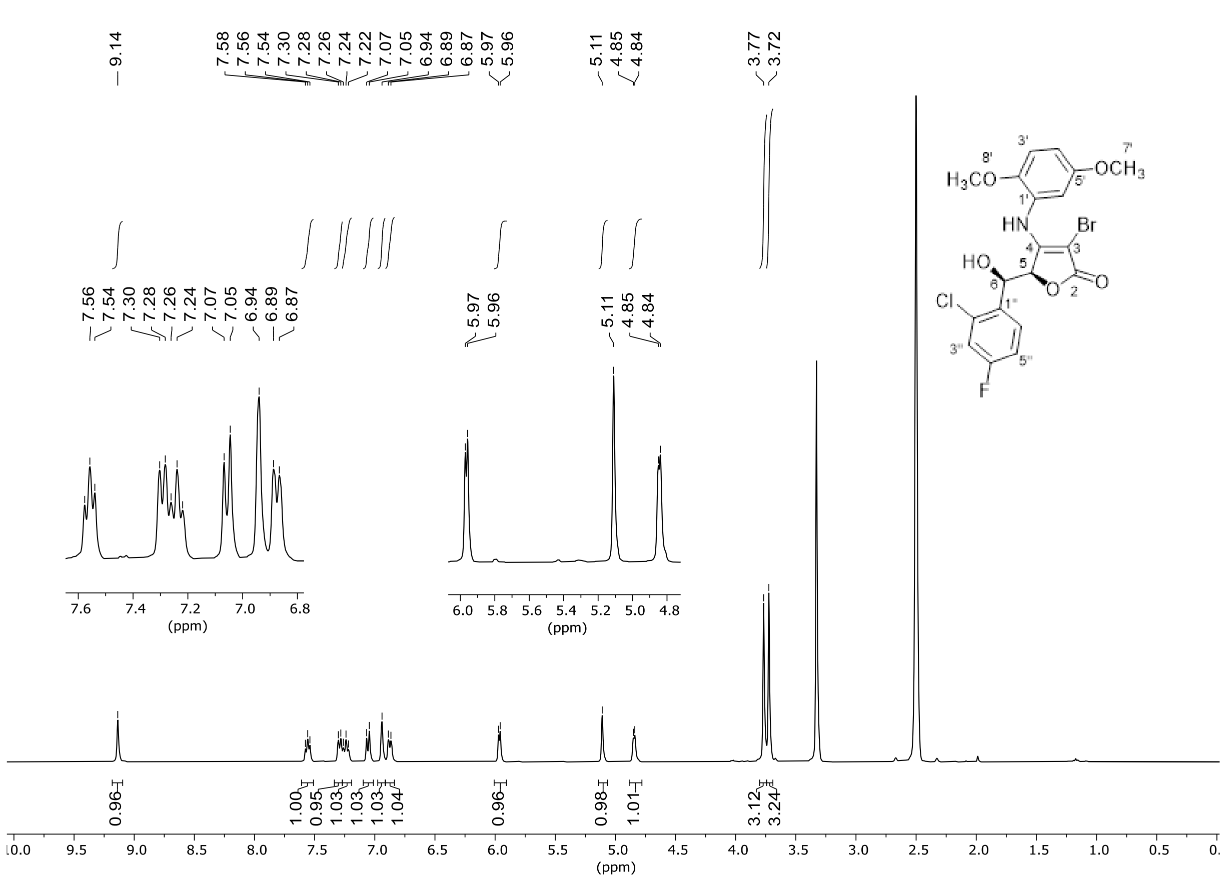

Figure S9: ^1H NMR (400 MHz, DMSO-d_6) of compound **17**.

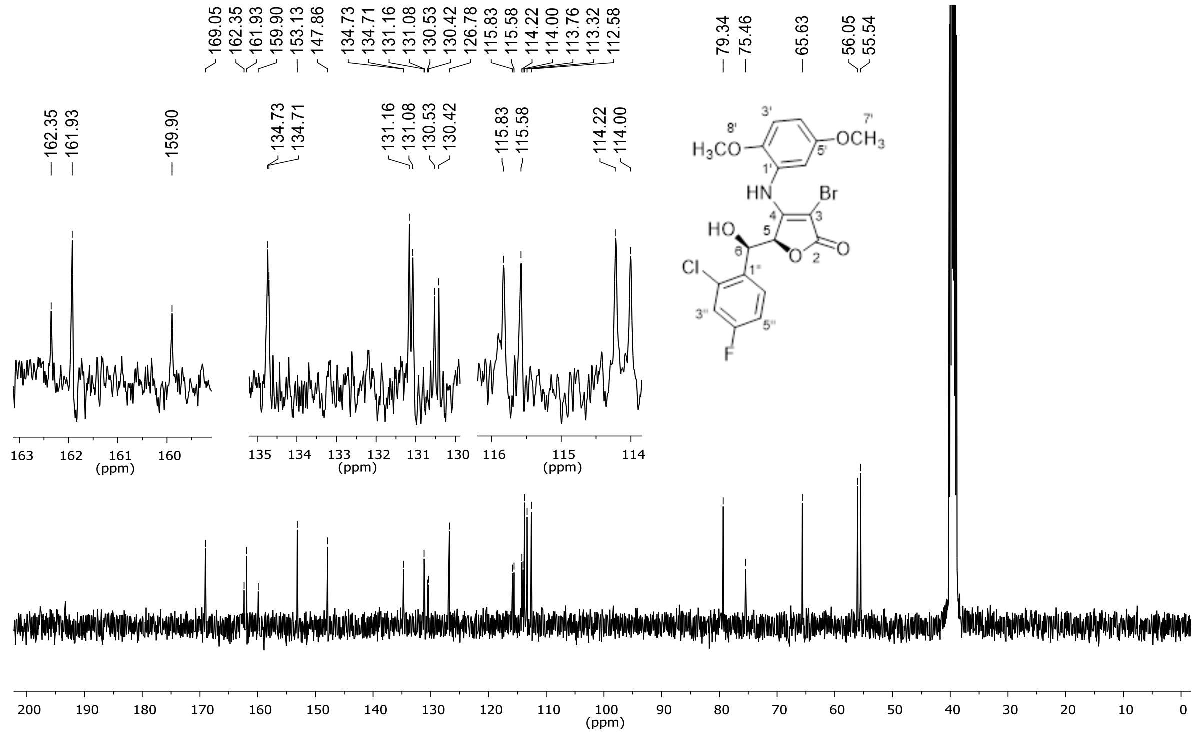

Figure S10: ^{13}C NMR (100 MHz, DMSO-d$_6$) of compound **17**.

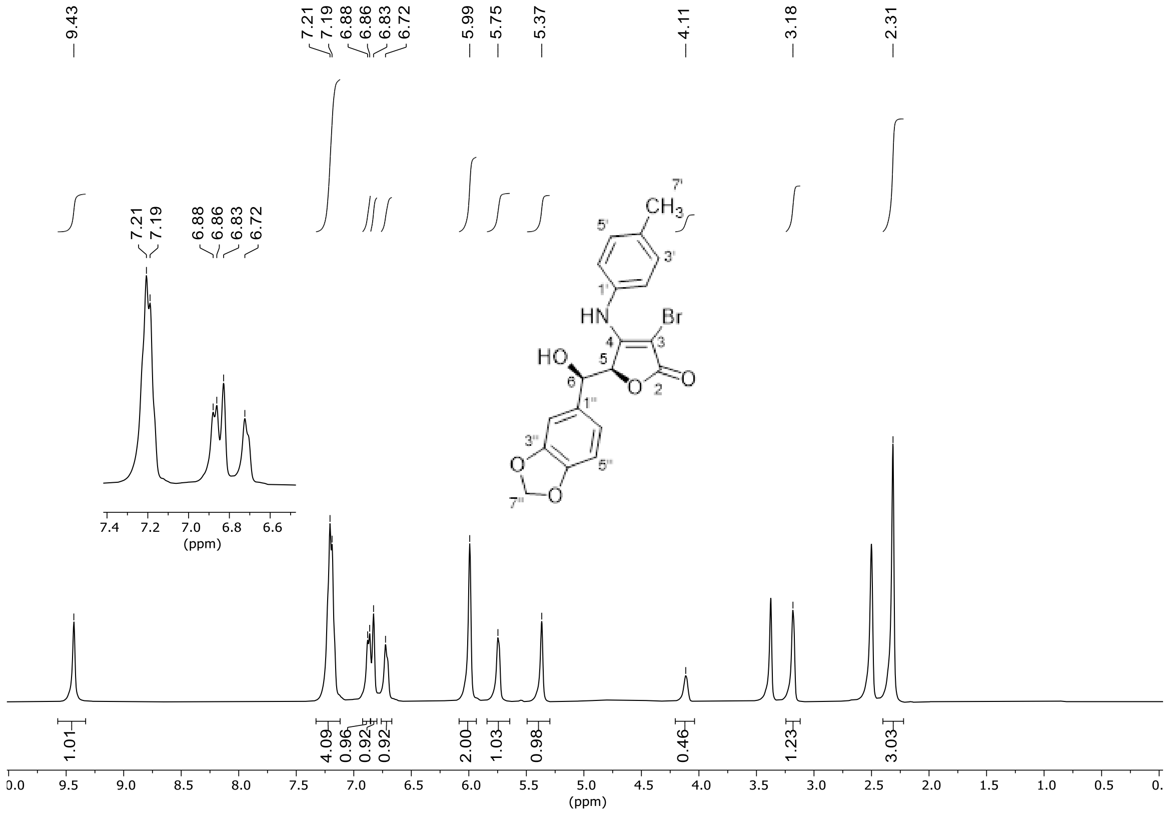

Figure S11: ^{1}H NMR (400 MHz, DMSO-d$_6$) of compound **18**.

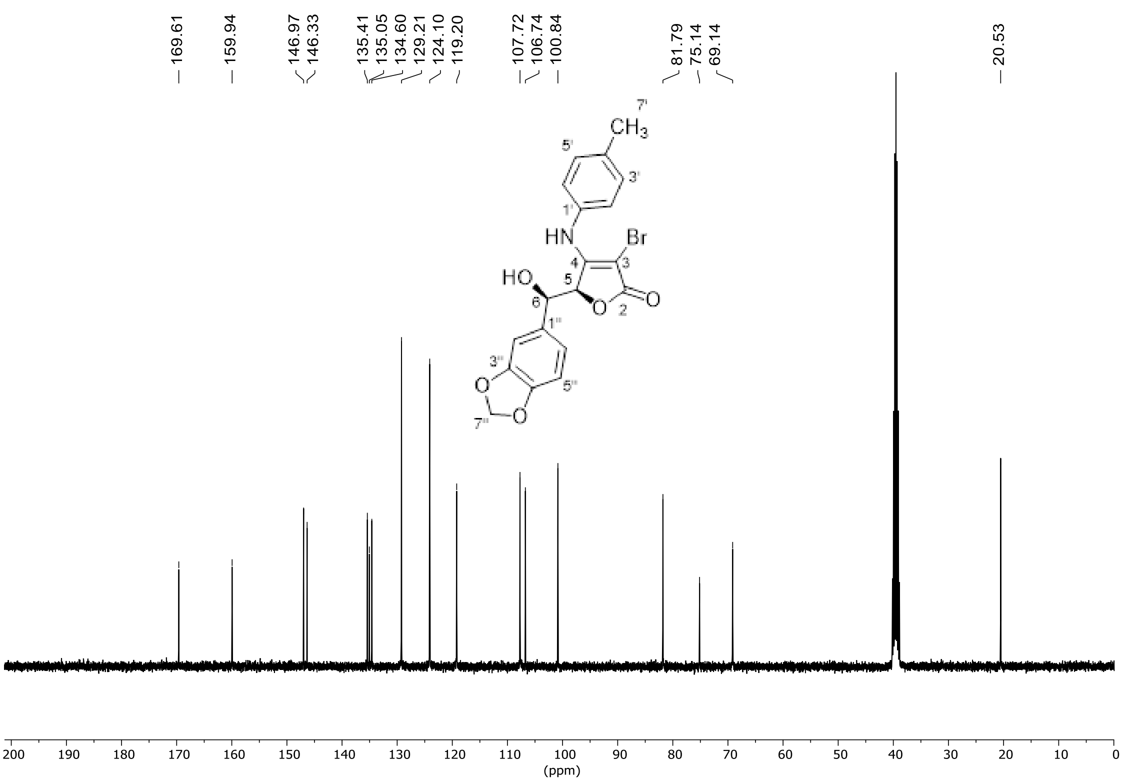

Figure S12: ^{13}C NMR (100 MHz, DMSO-d_6) of compound **18**.

5. Spectroscopic data for 4-amino-5-alkylidenebutenolide 20

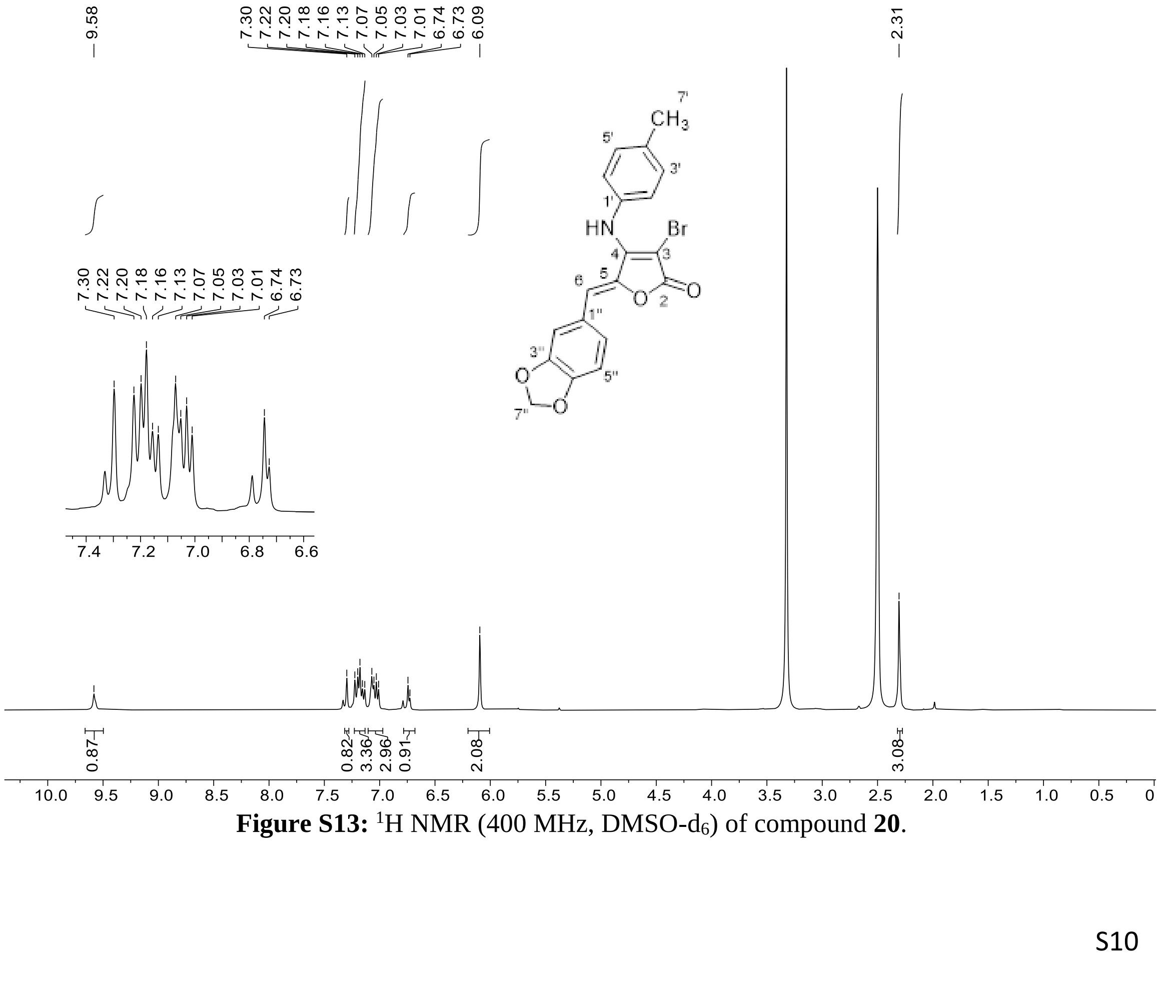

Figure S13: ^{1}H NMR (400 MHz, DMSO-d_6) of compound **20**.

6. Biological assays

Table S1: Effects of tetronamides compounds (50 μg/mL) on *E. coli* RP437 biofilm formation.

	Percentage of biomass (%)		
	**Total Growth**	**Bottom of the well**	**Total Biofilm**
Control	100.0 ± 0.0	100.0 ± 0.0	100.0 ± 0.0
6	114.4 ± 0.4	756.1 ± 153.0	185.7 ± 33.6
7	120.8 ± 5.8	722.2 ± 137.1	125.5 ± 46.8
8	76.1 ± 4.6	857.9 ± 279.2	131.6 ± 52.5
9	107.4 ± 1.9	1477.8 ± 519.3	228.6 ± 26.7
10	113.1 ± 5.1	1643.3 ± 556.4	234.5 ± 53.6
11	125.6 ± 4.5	2491.4 ± 851.1	367.3 ± 45.4
13	106.8 ± 3.0	230.3 ± 313.5	240.5 ± 30.0
14	123.7 ± 5.9	68.1 ± 33.3	49.1 ± 12.2
16	121.0 ± 1.2	1134.2 ± 427.6	146.7 ± 30.1
17	121.4 ± 1.0	113.5 ± 33.2	184.2 ± 15.0
18	116.3 ± 0.6	575.2 ± 303.2	190.8 ± 14.3
19	113.8 ± 0.7	79.3 ± 23.1	145.2 ± 34.0
20	116.3 ± 2.1	85.9 ± 41.6	32.0 ± 4.6
21	94.5±2.8	16.3 ± 47.8	108.4 ± 13.7

Table S2: Effect of tetronamides compounds (10 μg/mL) on AHL (AI-1) based quorum sensing, evaluated by measuring the bioluminescence of *V. harveyi* BB886.

	Vibrio harvey BB886	
	LUM/ CFU	%
**Control**	11.4	100.0 ± 0.0
6	4.3	36.3 ± 13.7
7	3.9	32.6 ± 17.9
8	3.8	32.8 ± 8.9
9	5.2	44.0 ± 17.4
10	4.9	40.9 ± 19.0
11	2.9	25.8 ± 0.6
13	1.1	9.5 ± 0.6
14	4.5	39.3 ± 0.5
16	1.3	11.8 ± 0.8
17	0.6	4.9 ± 1.0
18	2.7	24.4 ± 4.2
19	0.7	6.4 ± 0.9
20	16.2	149.8 ± 65.7
21	1.9	16.6 ± 2.2

Table S3: Effect of tetronamides compounds (10 µg/mL) on AI-2 based quorum sensing, evaluated by measuring the bioluminescence of *V. harveyi* BB170.

Vibrio harvey BB170		
	LUM/ CFU	%
**Control**	27.8	100.0 ± 0.0
6	5.5	20.8 ± 7.3
7	11.3	41.2 ± 3.7
8	4.8	17.6 ± 2.1
9	7.0	25.9 ± 7.1
10	12.5	47.0 ± 18.1
11	7.7	28.8 ± 9.5
13	3.5	13.1 ± 5.2
14	9.0	34.9 ± 20.3
16	19.3	71.1 ± 13.5
17	11.4	43.7 ± 22.0
18	25.6	96.4 ± 35.8
19	13.9	50.7 ± 7.1
20	11.7	42.8 ± 5.1
21	7.3	27.4 ± 10.5

Table S4: Effects of denigrins and precursors compounds (50 µg/mL) on *E. coli* RP437 biofilm formation.

	**Percentage of biomass (%)**		
	**Total Growth**	**Bottom of the well**	**Total Biofilm**
Control	100.0 ± 0.0	100.0 ± 0.0	100.0 ± 0.0
22	141.9 ± 3.3	249.2 ± 152.5	179.2 ± 98.0
23	129.9 ± 7.8	38.7 ± 25.2	48.2 ± 60.8
24	116.8 ± 5.8	27.9 ± 18.6	12.6 ± 3.1
25	135.1 ± 3.5	876.6 ± 315.2	94.0 ± 33.6
26	128.3 ± 4.7	15.4 ± 81.1	13.2 ± 15.6
27	111.8 ± 6.0	50.9 ± 17.4	58.1 ± 33.0

Table S5: Effect of denigrins and precursors (10 µg/mL) on AHL (AI-1) based quorum sensing, evaluated by measuring the bioluminescence of *V. harveyi* BB886.

Vibrio harvey BB886		
	LUM/ CFU	%
**Control**	**30.6**	**100.0 ± 0.0**
22	27.1	89.1 ± 17.9
23	25.5	82.3 ± 24.5
24	95.3	307.5 ± 111.24
25	198.5	654.4 ± 162.5
26	26.3	85.9 ± 3.8
27	358.5	1173.9 ± 220.3

Table S6: Effect of denigrins and precursors (10 μg/mL) on AI-2 based quorum sensing, evaluated by measuring the bioluminescence of *V. harveyi* BB170.

	Vibrio harvey BB170	
	LUM/ CFU	%
**Control**	**94.0**	**100.0 ± 0.0**
22	95.9	98.3 ± 22.2
23	9.8	10.6 ± 1.2
24	43.9	41.9 ± 24.0
25	81.4	91.2 ± 19.9
26	8.3	9.2 ± 1.7
27	92.9	103.6 ± 21.1

7. References

1. Karak, M.; Barbosa, L. C. A.; Acosta, J. A. M.; Sarotti, A. M.; Boukouvalas, J., Thermodynamically driven, syn-selective vinylogous aldol reaction of tetronamides. *Org. Biomol. Chem.* **2016,** *14* (21), 4897-4907.
2. Karak, M.; Barbosa, L. C. A.; Maltha, C. R. A.; Silva, T. M.; Boukouvalas, J., Palladium-catalyzed hydrodehalogenation of butenolides: An efficient and sustainable access to β-arylbutenolides. *Tetrahedron Lett.* **2017,** *58* (29), 2830-2834.
3. Karak, M.; Acosta, J. A. M.; Barbosa, L. C. A.; Sarotti, A. M.; da Silva, C. C.; Boukouvalas, J.; Martins, F. T., Substituent-modulated conformation and supramolecular assembly of tetronamides. *Cryst. Growth Des.* **2016,** *16* (10), 5798-5810.
4. Acosta, J. A.; Karak, M.; Barbosa, L. C.; Boukouvalas, J.; Straforini, A.; Forlani, G., Synthesis of new tetronamides displaying inhibitory activity against bloom-forming cyanobacteria. *Pest Manag. Sci.* **2020,** *76* (2), 779-788.
5. Karak, M.; Oishi, T.; Torikai, K., Synthesis of anti-tubercular marine alkaloids denigrins A and B. *Tetrahedron Lett.* **2018,** *59* (29), 2800-2803.
